# Airway Mucus Restricts Neisseria meningitidis Away from Nasopharyngeal Epithelial Cells and Protects the Mucosa from Inflammation

**DOI:** 10.1128/mSphere.00494-19

**Published:** 2019-12-04

**Authors:** Mathilde Audry, Catherine Robbe-Masselot, Jean-Philippe Barnier, Benoit Gachet, Bruno Saubaméa, Alain Schmitt, Sophia Schönherr-Hellec, Renaud Léonard, Xavier Nassif, Mathieu Coureuil

**Affiliations:** a INSERM, Unité U1151, Institut-Necker-Enfants-Malades, Paris, France; b Université de Paris, Faculté de Médecine, Paris, France; c CNRS, UMR 8253, Paris, France; d Université de Lille, CNRS, UMR8576-UGSF-Unité de Glycobiologie Structurale et Fonctionnelle, Lille, France; e Cellular and Molecular Imaging facility, INSERM US25, UMS3612 CNRS, Faculté de Pharmacie de Paris, Université de Paris, Paris, France; f Institut Cochin, INSERM U1016, Paris, France; g Assistance Publique–Hôpitaux de Paris, Hôpital Necker Enfants Malades, Paris, France; University of Michigan—Ann Arbor

**Keywords:** airway mucus, host-pathogen interaction, meningitis, nasopharynx, *Neisseria meningitidis*

## Abstract

Neisseria meningitidis is an inhabitant of the nasopharynx, from which it is transmitted from person to person or disseminates in blood and becomes a harmful pathogen. In this work, we addressed colonization of the nasopharyngeal niche by focusing on the interplay between meningococci and the airway mucus that lines the mucosa of the host. Using Calu-3 cells grown in air interface culture (cells grown with the apical domain facing air), we studied meningococcal colonization of the mucus and the host response. Our results suggested that N. meningitidis behaved like commensal bacteria in mucus, without interacting with human cells or actively transmigrating through the cell layer. As a result, type IV pili do not play a role in this model, and meningococci did not trigger a strong innate immune response from the Calu-3 cells. Finally, we have shown that this model is suitable for studying interaction of N. meningitidis with other bacteria living in the nasopharynx and that Streptococcus mitis, but not Moraxella catarrhalis, can promote meningococcal growth in this model.

**IMPORTANCE** N. meningitidis is transmitted from person to person by aerosol droplets produced by breathing, talking, or coughing or by direct contact with a contaminated fluid. The natural reservoir of N. meningitidis is the human nasopharynx mucosa, located at the back of the nose and above the oropharynx. The means by which meningococci cross the nasopharyngeal wall is still under debate, due to the lack of a convenient and relevant model mimicking the nasopharyngeal niche. Here, we took advantage of Calu-3 cells grown in air interface culture to study how meningococci colonize the nasopharyngeal niche. We report that the airway mucus is both a niche for meningococcal growth and a protective barrier against N. meningitidis infection. As such, N. meningitidis behaves like commensal bacteria and is unlikely to induce infection without an external trigger.

## Abstract (precis)

N. meningitidis is transmitted from person to person by aerosol droplets produced by breathing, talking, or coughing or by direct contact with a contaminated fluid. The natural reservoir of N. meningitidis is the human nasopharynx mucosa, located at the back of the nose and above the oropharynx. The means by which meningococci cross the nasopharyngeal wall is still under debate, due to the lack of a convenient and relevant model mimicking the nasopharyngeal niche. Here, we took advantage of Calu-3 cells grown in air interface culture to study how meningococci colonize the nasopharyngeal niche. We report that the airway mucus is both a niche for meningococcal growth and a protective barrier against N. meningitidis infection. As such, N. meningitidis behaves like commensal bacteria and is unlikely to induce infection without an external trigger.

## INTRODUCTION

Neisseria meningitidis (meningococcus) is a Gram-negative bacterium that normally resides asymptomatically in the human nasopharynx. For unknown reasons, it may cross the epithelial barrier and proliferate in the bloodstream where it becomes one of the most harmful pathogens. N. meningitidis effectively adheres to the endothelial cells lining the lumen of blood vessels (1). From there, bacteria proliferate and cause blood vessel dysfunction (2–6) responsible for the rapid progression of septic shock, leading in the worst case to purpura fulminans, an acute systemic inflammatory response associated with intravascular coagulation and tissue necrosis. N. meningitidis can also cross the blood-brain barrier and cause cerebrospinal meningitis (7, 8).

N. meningitidis is transmitted from person to person by aerosol droplets produced by breathing, talking, or coughing or by direct contact with a contaminated fluid. The natural reservoir of N. meningitidis is the human nasopharynx mucosa, located at the back of the nose and above the oropharynx. There, the bacteria encounter a rich microbiota (9–11) that continuously undergoes changes with age and upon upper respiratory infections (12, 13). The nasopharynx is lined with two main types of epithelium: a pluristratified squamous epithelium that covers 60% of the nasopharynx and a columnar respiratory epithelium (14, 15). In the respiratory tract, cells are protected by a 10- to 12-μm-thick two-layer surface liquid formed by a low-viscosity periciliary liquid (PCL) in contact with the cells and a high-viscosity mucus facing the lumen that retains bacteria, inhaled particles, and cell debris (outer mucus) (16, 17). The PCL facilitates ciliary beating that allows effective mucociliary clearance at 6.9 ± 0.7 mm/min (18). By constantly transporting mucus from the lower respiratory tract to the pharynx from where it is swallowed, this mechanism is considered the main defense against microorganisms and particles. The mucus layer in which commensal bacteria are restricted is a thick gel formed by mucins and contains many antimicrobial proteins and peptides such as IgA, lysozyme, lactoferrin, and human defensins (19–21). Mucins are a family of at least 22 high-molecular-weight glycoproteins divided into two classes: membrane-associated mucins that are produced by any epithelial cell and gel-forming mucins produced by goblet cells and submucosal glands. In the respiratory tract, the mucus layer is mainly composed of MUC5AC and MUC5B. Their expression is tightly regulated and responds to bacterial infections and to a variety of respiratory tract diseases (17).

The interaction of N. meningitidis with epithelial cells has been the subject of numerous studies over the past 4 decades. However, the means by which meningococci cross the nasopharyngeal wall is still under debate. This may be due to the lack of a convenient and relevant model mimicking the nasopharyngeal niche. Most of the previous studies, addressing the adhesion-dependent interaction of N. meningitidis with intestinal and respiratory tract epithelial cells, have been performed on cells cultured in liquid media such as RPMI and Dulbecco modified Eagle medium (DMEM). These first studies gave rise to the concept of type IV pilus- and/or Opa/Opc-mediated cell colonization. In such a model, meningococci interact with epithelial cells through their type IV pili and form highly proliferative microcolonies that eventually cross the epithelial barrier (22–28), while Opa and Opc proteins are involved in an active internalization process that is supposed to promote the translocation of bacteria through the cell monolayer (29–31). Each of these works demonstrated a close interaction of N. meningitidis with human epithelial cells. Over the last decade, few studies have focused on the translocation of N. meningitidis through the epithelial layer. The work of T. C. Sutherland et al. in 2010 (32) addresses this question by using Calu-3 human bronchial epithelial cells, a respiratory tract cellular model that can be fully differentiated into a polarized epithelium. Although the authors worked with cells infected in liquid-covered culture (LCC), they proposed using Calu-3 cells in an air interface culture (AIC), a model in which the cells are grown with the apical domain facing air. They finally concluded that N. meningitidis may cross the epithelial layer by the transcellular route using type IV pili. Meanwhile, Barrile et al. have shown, using Calu-3 cells grown in LCC, that meningococci may be internalized and transported to the basal domain by subverting the intracellular traffic of the host cells (33). However, they have also shown that the translocation of bacteria was fully inhibited in highly polarized cells cultured for 18 days.

In addition to these works, a series of *ex vivo* experiments were conducted between 1980 and 1995 using organ cultures instead of immortalized cells (34–37). The authors have observed a direct interaction between meningococci and explant epithelial cells. This has been associated with the loss of cilia and, for some explants, with the internalization of bacteria in epithelial cells. However, in each of these experiments, the explants were immersed in liquid medium, a protocol that may have altered cell morphology and disrupted the mucus barrier.

The study of meningococcal colonization of the human upper respiratory tract has been hampered by the lack of relevant models. In this work, we took advantage of Calu-3 cells grown in AIC (38) to study how meningococci colonize the nasopharyngeal niche. Infection of Calu-3 cells revealed the dependence of N. meningitidis on mucus in this model. Our results suggest that the mucus protects meningococci against death associated with desiccation and supports the growth of bacteria. We have shown that the mucus layer sequestered bacteria and that a direct interaction of bacteria with the epithelial cells was rarely observed. Bacteria grew without triggering a strong innate immune response from the Calu-3 cells. Embedded in the mucus, meningococci were protected and fed, and expressed lower levels of adhesins but a high level of iron transporters, while type IV pili were not necessary for colonization. Finally, we evaluated the effect of Streptococcus mitis and Moraxella catarrhalis colonization, two bacteria classically present in the nasopharynx mucosa, on the growth of N. meningitidis (39–41) and showed that cocolonization of N. meningitidis with S. mitis can promote meningococcal growth.

## RESULTS

### Meningococci require mucus production to colonize cells cultured at the air-liquid interface.

**FIG 1:**
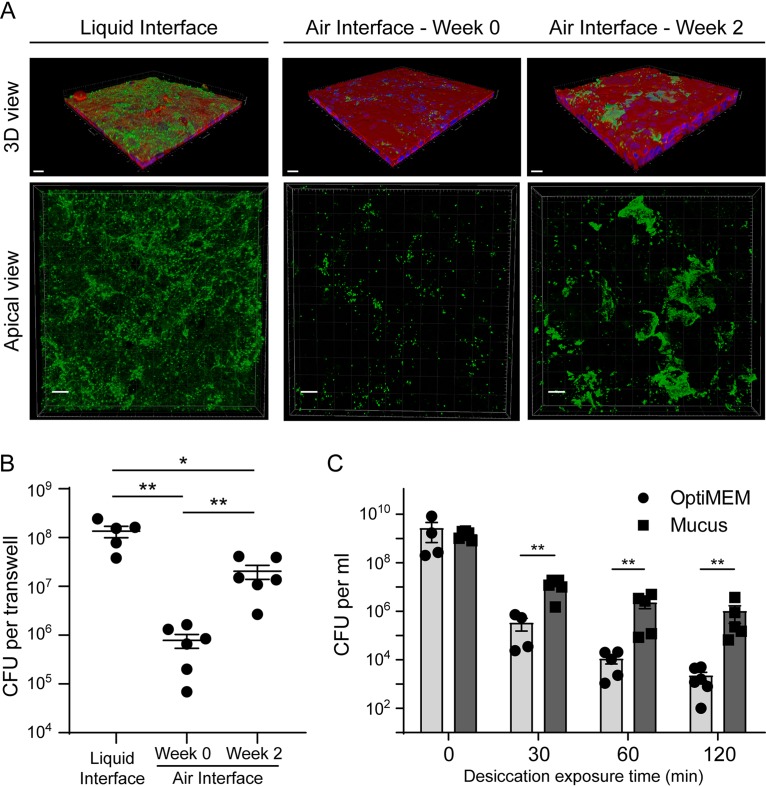
N. meningitidis proliferation in an air interface culture model. (A) Confocal 3D reconstructions showing N. meningitidis proliferation 24 h after infection of Calu-3 cells. Meningococci were labeled with anti-2C43 antibody (green). Cells were stained with Alexa Fluor-conjugated phalloidin (red). Nuclei were stained with DAPI (blue). Bars, 20 μm. (B) Number of CFU of N. meningitidis per well 24 h after infection (10^6^ bacteria). Data were expressed as mean values of CFU per filter ± standard errors of the means (SEM) (error bars). Statistical analysis was performed by one-way ANOVA on at least five filters from three independent experiments. Values that are statistically significantly different are indicated by bars and asterisks as follows: *, *P* < 0.05; **, *P* < 0.01. (C) Desiccation assay. Plates were coated with the mucus obtained from Calu-3 cells or with the culture medium as a control. Bacteria were then grown overnight in these wells containing culture media. The medium was then gently removed. Bacteria were dried for 0, 30, 60, or 120 min. The number of CFU was then assessed. Data were expressed as mean values of CFU per milliliter ± SEM. Statistical analysis was performed by Student’s *t* test on at least four wells from two independent experiments. Values that are statistically significantly different are indicated by bars and asterisks as follows: **, *P* < 0.01.

To study the colonization of the human upper respiratory tract by Neisseria meningitidis, we used Calu-3 cells grown on 0.4-μm-pore membrane under AIC. Cells maintained for a few days in AIC (week 0) formed a monostratified epithelium with only a few spots of mucus on the cell surface (see Fig. S1A in the supplemental material). We observed pseudostratified Calu-3 cells covered with a mucus layer after 2 weeks of AIC. After 3 weeks of AIC, the epithelium appeared pluristratified with a thick layer of mucus above the cells (Fig. S1A). Cells grown for 2 weeks in AIC also revealed the presence of tight junctions, microvilli, and mucus-producing cells (Fig. S1B). Two weeks after AIC, mucus thickness has been estimated at 30.6 ± 5.6 μm (Fig. S1C). We first considered whether mucus may influence the growth of meningococci on Calu-3 cells grown in AIC for 2 days or 2 weeks (AIC W_0_ and AIC W_2_, respectively). We added 1 × 10^6^ meningococci (strain 2C4.3) over the cells and assessed epithelial colonization by confocal imaging and quantitative culture (CFU counts) 24 h after infection. These results were compared to those obtained after infection of Calu-3 cells cultured under liquid-covered culture (LCC). We observed a dramatic decrease in bacterial proliferation 24 h after infection of AIC W_0_ cultured cells compared to LCC cultured cells (1.35 × 10^8^ ± 0.35 × 10^8^ bacteria per well in LCC; 0.75 × 10^6^ ± 0.25 × 10^6^ bacteria per well in AIC W_0_). This inhibition is less pronounced in AIC W_2_ cultured cells that produced mucus (2 × 10^7^ ± 0.64 × 10^7^ bacteria in AIC W_2_). In addition, in LCC, bacteria are evenly distributed at the surface of the mucus (Fig. 1A and B). These results suggest that LCC is more favorable to bacterial growth than AIC.

10.1128/mSphere.00494-19.1 FIG S1 Calu-3 cells grown using AIC. (A) Confocal 3D reconstructions of living Calu-3 cells showing accumulation of the mucus after 2 weeks of culture in AIC. The mucus was labeled with Alexa Fluor-conjugated dextran (cyan), and cells were stained with Cell Trace Calcein Red Orange, AM (red). Bar, 20 μm. (B) Transmission electron microscopy images (transversal section). Tight junction (asterisk), mucin-containing vesicles (arrow), microvilli (μV), and mucus (M) are indicated. Bars, 2 μm. (C) Measurement of mucus thickness after 2 or 3 weeks of culture in AIC. Measures were obtained from analysis of confocal images, using ImageJ. Mucus thickness was expressed in microns (mean ± SEM). Statistical analysis was performed by Mann-Whitney U test (20 measures were performed on two representative *x-z* and *x-y* images). Values for mucus thickness at week 2 and week 3 were statistically significantly different (*P < *0.0001). Download FIG S1, TIF file, 1.8 MB. Copyright © 2019 Audry et al. 2019 Audry et al. This content is distributed under the terms of the Creative Commons Attribution 4.0 International license.

The mucus layer is known to be a poor nutritive medium that limits the growth of many commensal and pathogenic bacteria. It is a highly hydrated gel that also protects the cell surface from desiccation. We therefore aimed at determining whether the mucus layer could protect N. meningitidis against desiccation in an abiotic surface colonization model (Fig. 1C). We infected plastic wells coated with purified and dried mucus for 24 h to allow bacteria to adhere and biofilm to form at the bottom of wells (see Materials and Methods). It is important to note that the mucus did not favor the growth of bacteria. We counted the same number of CFU, before desiccation, in both control wells and mucus-coated wells (1.7 × 10^7^ and 1.4 × 10^7^ CFU, respectively). The day of the experiment, we removed the culture media and incubated adherent bacteria under a laminar flow hood for 30, 60, and 120 min. In this condition, meningococci were particularly sensitive to desiccation. In the control wells, the number of living bacteria was reduced by 5.6 × 10^3^-fold at 30 min and 7.9 × 10^5^-fold at 120 min after the beginning of the experiment. Conversely, in mucus-covered wells, the number of living bacteria was reduced by 110-fold at 30 min and 1.46 × 10^3^-fold at 120 min after the beginning of the experiment. Overall, our results indicate that the mucus layer of Calu-3 cells cultured in AIC is likely to protect bacteria from desiccation.

### Meningococci are restricted in the mucus layer and do not cross the epithelium.

**FIG 2:**
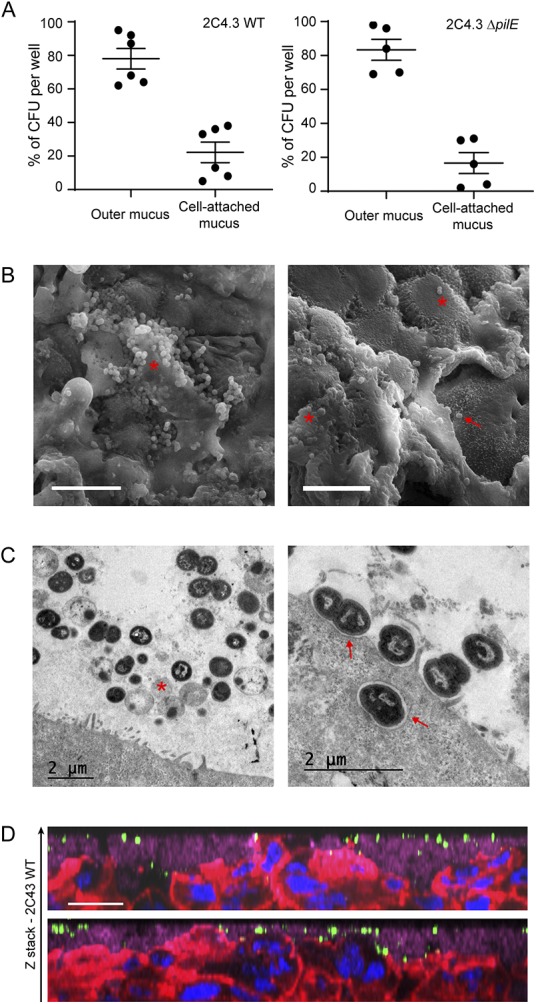
N. meningitidis colonizes the outer mucus. Calu-3 cells grown in AIC were infected for 24 h with 10^6^ bacteria. (A) After Calu-3 cells were infected with wild-type (WT) or Δ*pilE* mutant of N. meningitidis strain 2C4.3, the outer layer of the mucus (outer mucus) was dissociated from the cell-attached mucus using *N*-acetylcysteine. Bacterial load in the *N*-acetylcysteine fraction or the cell-attached fraction was determined. Data were expressed as mean percentages of CFU ± SEM for at least five filters from three independent experiments. (B) Scanning electron microscopy images showing bacteria trapped in the mucus. Bacteria trapped in mucus (red asterisk) and bacteria that directly interact with Calu-3 cells (red arrow) are indicated. Bars, 10 μm. (C) Transmission electron microscopy images showing bacteria in the mucus (left) or in contact with cells (right). A dying bacterium (red asterisk) and bacteria adherent to Calu-3 cells (red arrows) are indicated. (D) Z-stack from confocal 3D reconstruction of two different Calu-3 cell layers infected with N. meningitidis. Calu-3 cell layers were fixed and immunostained with anti-2C4.3 antibody (green) and anti-MUC5AC antibody (purple). Cells were stained with A546-phalloidin (red), and nuclei were stained with DAPI (blue). Bar, 20 μm.

**FIG 3:**
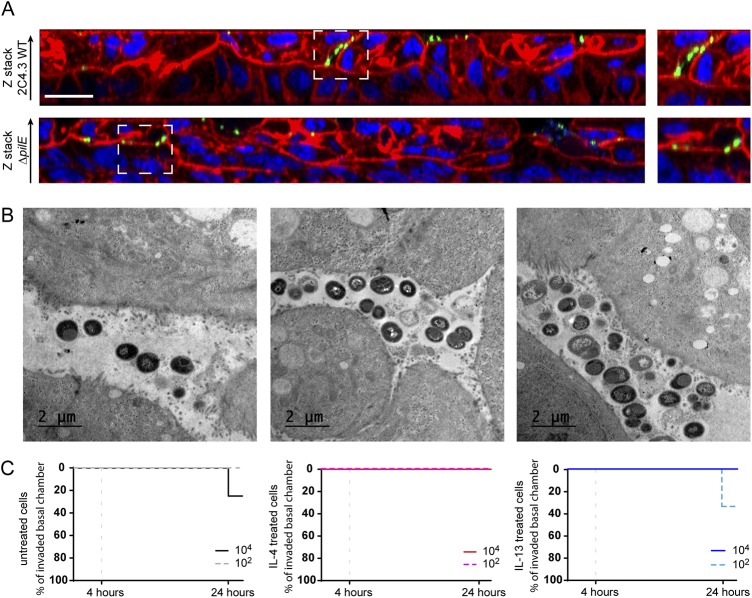
N. meningitidis do not cross the epithelial layer. (A) Z-stack from confocal 3D reconstruction of Calu-3 cell layer infected 24 h with 10^6^ bacteria of the wild-type strain (2C4.3 WT) or the strain defective for type IV pili (2C4.3 Δ*pilE*). The Calu-3 cell layer was fixed and immunostained with anti-2C4.3 antibody (green). Cells were stained with Alexa Fluor-conjugated phalloidin (red), and nuclei were stained with DAPI (blue). Bar, 20 μm. (B) Transmission electron microscopy images (longitudinal sections) of Calu-3 cell layer infected for 24 h with 10^6^ wild-type meningococci. (C) Kaplan-Meier plots showing the traversal of wild-type N. meningitidis across the Calu-3 cell layer. Data were expressed as percentages of invaded basal chamber. For untreated cells, eight filters were infected with 10^4^ or 10^2^ bacteria, and three independent experiments were performed. For IL-4- or IL-13-treated cells, four filters were infected with 10^4^ or 10^2^ bacteria, and two independent experiments were performed.

During LCC infection, bacteria readily adhere to human cells and induce host cell signaling, leading to the recruitment of ezrin and actin and the formation of membrane protrusions (42, 43). Conversely, during AIC infection, as would be the case during colonization of nasopharyngeal mucus, bacteria are deposited on the mucus layer that protects the cells. We therefore studied how N. meningitidis interact with the epithelium grown in AIC. First, we compared the number of CFU recovered from the outer mucus with a fraction containing both cells and the cell-attached mucus. The cells were infected with 1 × 10^6^ wild-type meningococci or its nonpiliated derivative (*pilE* mutant; Δ*pilE*), which is unable to adhere to human cells (24). Up to 80% of wild-type meningococci or Δ*pilE* meningococci have been recovered in the outer layer of the mucus, which means that N. meningitidis penetrates this layer only slightly (Fig. 2A). Interestingly, the same amount of wild-type and Δ*pilE* meningococci was collected in the fraction containing cells and the cell-attached mucus. To better characterize the infection of Calu-3 cells grown using AIC and determine whether the bacteria interacted with the cells, we visualized infected cells by transmission and scanning electron microscopy (TEM and SEM). We found that most bacteria were trapped in the mucus (Fig. 2B and C) and organized into small aggregates of living and dying meningococci, according to cell morphology. Few bacteria were found in direct contact with the plasma membranes of Calu-3 cells, and we rarely detected membrane protrusions near bacteria, in contrast to what has been observed previously when cells were infected in LCC (43). We detected few internalized bacteria, despite analysis of four different longitudinally cut cell layers. These bacteria were in the vicinity of the apical plasma membrane (Fig. 2D) or already digested in a vacuole (Fig. S2). We then studied the infected Calu-3 cells by confocal microscopy. Again, most bacteria were detected in the mucus, stained with anti-MUC5AC antibody. We also observed both by confocal microscopy and TEM, microcolonies of bacteria trapped between epithelial cells in cavities in the top part of the cell layer (Fig. 3A and B). However, no bacteria were observed in the basal part of the epithelium as would have been expected if the bacteria had passed through it. In addition, nonpiliated Δ*pilE* mutant showed the same spatial location as the wild-type strain, suggesting that type IV pili have no role in the location of bacteria in the mucus (Fig. 3A).

10.1128/mSphere.00494-19.2 FIG S2 Dying and living N. meningitidis. Transmission electron microscopy images (longitudinal section) showing dying and living bacteria inside the mucus (A) and dying bacteria inside a cell (B). Dead bacteria (DB) and living bacteria (LB) are indicated. Bars, 1 μm. Download FIG S2, TIF file, 1.6 MB. Copyright © 2019 Audry et al. 2019 Audry et al. This content is distributed under the terms of the Creative Commons Attribution 4.0 International license.

On the basis of this observation, we studied the translocation of meningococci through the epithelial cell layer. We first grew Calu-3 cells in AIC using 3-μm-pore membranes instead of 0.4-μm-pore membranes. We chose to infect Calu-3 cells with 10^2^ or 10^4^ bacteria, and we first confirmed the proliferation of N. meningitidis under these conditions (Fig. S3A and B). Regardless of the inoculum, the number of colonizing bacteria at 24 h, 10^7^ CFU, was similar. We then studied the translocation of N. meningitidis from the mucus to the basal chamber, by plating the basal media on agar plates, at 4 and 24 h after infection. We considered translocation positive when at least one CFU has been recovered from the basal chamber. Interestingly, 24 h after infection, we detected only 2 out of 16 colonized basal chambers in total, and no bacteria were recovered in the basal chambers 4 h after infection (Fig. 3C). Using this model, we then evaluated the effect of interleukin-4 (IL-4) or IL-13, two cytokines known to be involved in pharyngeal inflammation (44), on the translocation of N. meningitidis. The cells were treated 24 h with 5 ng/ml IL-4 or IL-13 as previously described (44) (Fig. 3C). As expected, the treatment of Calu-3 cells with IL-4 or IL-13 resulted in a twofold decrease in transepithelial electric resistance (TEER) (165.9 ± 20.02 Ω · cm^2^ and 155.3 ± 11.58 Ω · cm^2^, respectively; Fig. S3C). However, this has not been associated with an increased traversal of the cell layer by N. meningitidis (Fig. 3C). Our results indicate that meningococci are likely to colonize the outer layer of mucus, from which bacteria can reach the cell-attached mucus but rarely come into contact with cells or cross the epithelial layer cultured in AIC.

10.1128/mSphere.00494-19.3 FIG S3 Culture of Calu-3 cells in the presence of IL-4 and IL-13 and infection of Calu-3 cells. (A) Number of meningococci 24 h after infection of Calu-3 cells, grown in AIC, with 10^2^, 10^4^, or 10^6^ bacteria. Data were expressed as mean value of CFU per filter ± SEM for six filters from two independent experiments. (B) Confocal 3D reconstructions showing N. meningitidis proliferation. Twenty-four hours after infection of Calu-3 cells with 10^2^, 10^4^, or 10^6^ bacteria, the cells were fixed and immunostained with anti-2C4.3 antibody (green). Cells were stained using Alexa Fluor-conjugated phalloidin (in red). Nuclei were stained with DAPI (blue). Bar, 20 μm. (C) Measurement of the TEER of Calu-3 cell layer cultured in AIC using 0.4-μm-pore membrane or 3-μm-pore membrane, with or without the addition of 5 ng/ml IL-4 or IL-13 for 24 h. TEER was expressed as mean ohms · cm^2^ ± SEM. Statistical analysis was performed by one way-ANOVA (for 0.4- versus 3- μm-pore membrane, three filters; for control versus IL-4 or IL-13 treated, four filters from two independent experiments). Values that are statistically significantly different are indicated by asterisks as follows: ***, *P* < 0.001; ** *P* < 0.01. Download FIG S3, TIF file, 1.1 MB. Copyright © 2019 Audry et al. 2019 Audry et al. This content is distributed under the terms of the Creative Commons Attribution 4.0 International license.

### Expression of meningococcal virulence factors during AIC infection.

**FIG 4:**
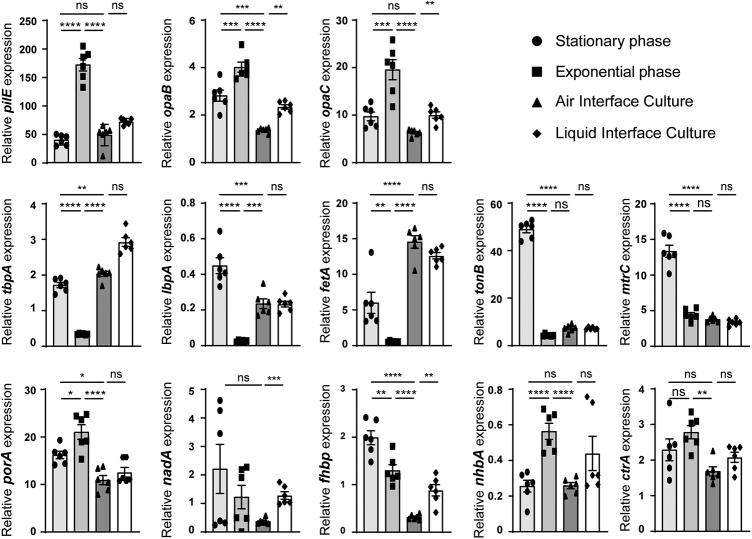
Expression of virulence factors. Total RNA obtained from a 3-h or 24-h broth culture or harvested after 24 h of infection of Calu-3 cells using AIC or LCC was prepared. Expression of the *pilE*, *opaB*, *opaC*, *tbpA*, *lbpA*, *fetA*, *tonB*, *mtrC*, *porA*, *nadA*, *fhbp*, *nhbA*, and *ctrA* genes was analyzed by quantitative RT-PCR. Gene expression was normalized to that of *pgm* and expressed as relative expression ± SEM. Statistical analysis was performed by one-way ANOVA on two independent experiments in triplicate. Values that are statistically significantly different are indicated by asterisks as follows: ****, *P* < 0.0001; ***, *P* < 0.001; **, *P* < 0.01; *, *P* < 0.05. Values that are not significantly different (ns) are indicated.

Our results suggest that, on mucosa, meningococci are likely to live trapped in the mucus. Under these conditions, it is likely that meningococci regulate the expression of their genes differently than in broth. We therefore characterized the relative expression of genes known to be involved in mucosal colonization. We have focused on the expression of genes encoding adhesion factors: *pilE*, *opaB*, *opaC*, and *nhbA*; genes encoding proteins involved in iron acquisition: *tbpA*, *lbpA*, *fetA*, and *tonB*; *mtrC* encoding the first gene of the *mtrCDE* operon that is involved in drug efflux; *ctrA* that codes for the capsular transport protein A; and the four genes coding for targets of the meningitidis B (MenB) vaccine, *porA*, *fhbp*, *nadA*, and *nhbA*. We compared the expression of these genes during AIC infection to their expression during the exponential and stationary phase of growth in broth (Fig. 4).

These genes followed different expression profiles. Expression of the adhesion factors (*pilE*, *opaB*, *opaC*, and *nhbA*) are comparable between the stationary phase of growth in broth and the infection of Calu-3 cells for 24 h. In contrast, expression of *pilE*, *opaB*, *opaC*, and *nhbA* in AIC was decreased with respect to the exponential phase of growth (AIC/exponential phase, 0.28, 0.34, 0.33, and 0.47, respectively). The three iron transporters tested (*tbpA*, *lbpA*, and *fetA*) were strongly expressed in AIC compared to the exponential phase of growth (AIC/exponential phase, 6.25, 10, and 27, respectively).

It is noteworthy that *fhbp*, *tonB*, and *mtrC* expression were weak during AIC infection. Their expressions were 6.3-, 6.7-, and 3.5-fold less in the mucus of Calu-3 cells than in the stationary phase of growth. *porA* expression is reduced in AIC compared to both the stationary phase and the exponential phase of growth (AIC/stationary phase, 0.67; AIC/exponential phase, 0.51). No major difference in the expression of *nadA* and *ctrA* was observed between the tested conditions. Overall, 9 out of the 13 tested genes appeared to follow the same pattern of expression in AIC as in the stationary phase of growth. We then addressed the impact on gene expression of mucus-bacterium interaction, compared to cell-bacterium interaction. Cells grown in LCC were infected for 24 h, and gene expression was quantified as described above. Interestingly, 9 out of the 13 genes followed the same expression profile in AIC and LCC. Only *opaB*, *opaC*, *nadA*, and *fhbp* expression were significantly increased during LCC infection compared to AIC infection. These results suggest that, 24 h after infection, the meningococcus do not recognize the mucus or the cells as a trigger for gene expression.

### Meningococci trigger less inflammation in the AIC condition.

**FIG 5:**
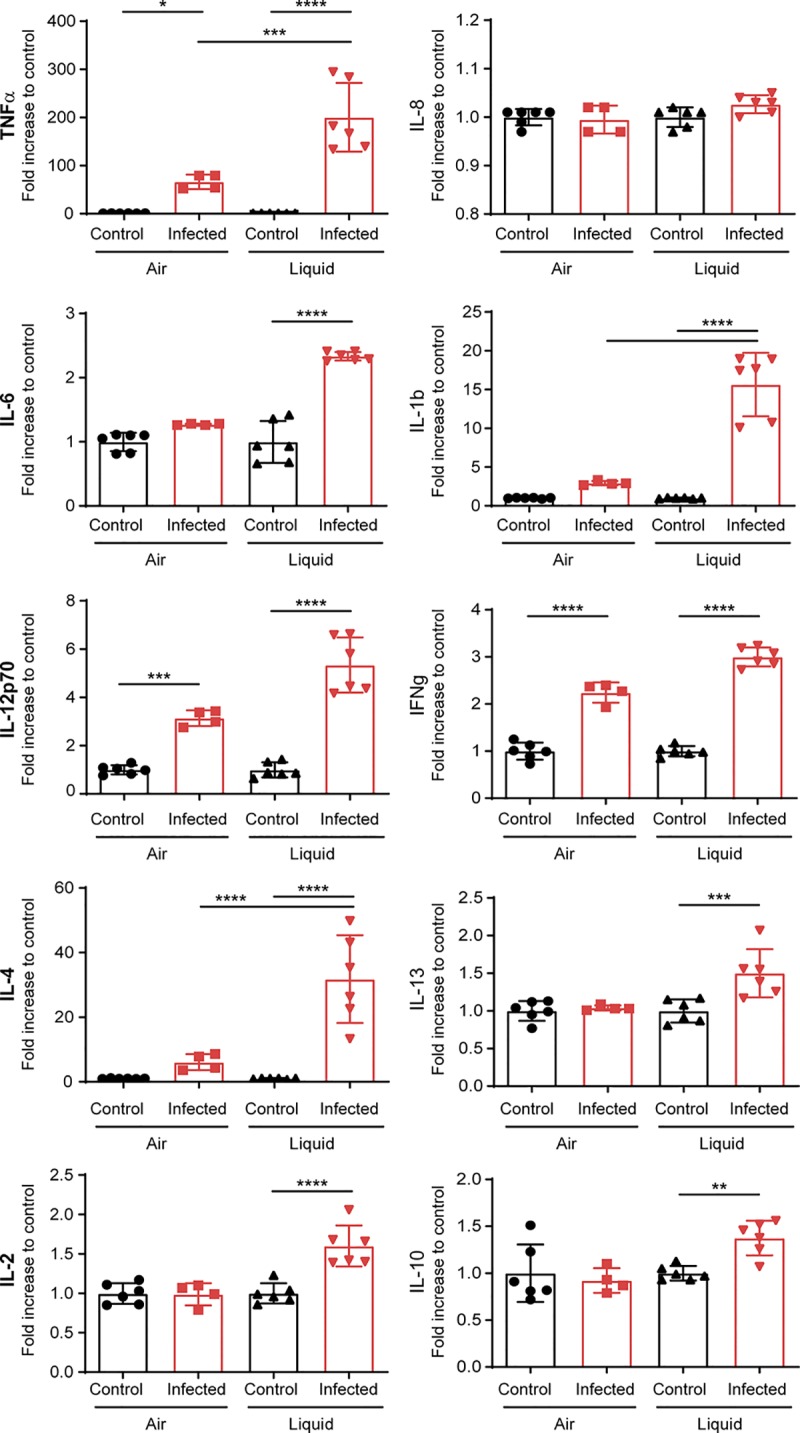
Cytokine expression by infected Calu-3 cells grown in AIC and LCC. Cytokine secretion was investigated in the mucus of noninfected and infected Calu-3 cells in AIC for 24 h or in the supernatant of noninfected and infected Calu-3 cells in LCC for 24 h. Data were expressed as means ± SEM of the fold increase between infected and noninfected conditions. Statistical analysis was performed by one-way ANOVA on at least two filters read in duplicate. Values that are statistically significantly different are indicated by asterisks as follows: ****, *P* < 0.0001; ***, *P* < 0.001; **, *P* < 0.01; *, *P* < 0.05.

We then addressed the impact of meningococcal colonization on the innate immune response of Calu-3 cells grown in AIC or LCC. We therefore measured the release of 10 pro- or anti-inflammatory cytokines 24 h after infection in comparison to the basal release of these cytokines by Calu-3 cells after 24 h of culture without bacteria (Fig. 5). We observed that after infection, three cytokines were produced in larger amounts under LCC infection than AIC infection: IL-1β showed 2.9-fold increase under AIC versus 15.6-fold increase under LCC; tumor necrosis factor alpha (TNF-α) showed 66.4-fold increase under AIC versus 200.4-fold increase under LCC; IL-4 showed 6-fold increase under AIC versus 31.8-fold increase under LCC. A moderate release of four other cytokines was detected only under LCC: IL-10, IL-13, IL-2, and IL-6 (1.4-fold increase, 1.5-fold increase, 1.6-fold increase, and 2.33-fold increase, respectively). Finally, gamma interferon (IFN-γ) secretion was increased by 2-fold under AIC versus 3-fold under LCC, and IL-12 secretion was increased by 3.1-fold under AIC versus 5.1-fold under LCC. Altogether, the proinflammatory response assessed by cytokine production, appeared to be higher in the LCC model compared to the AIC model. In addition, we did not observe any secretion of IL-2, IL-10, IL-13, and IL-6 during an AIC infection.

### Streptococcus mitis colonization of Calu-3 cells promotes N. meningitidis growth.

**FIG 6:**
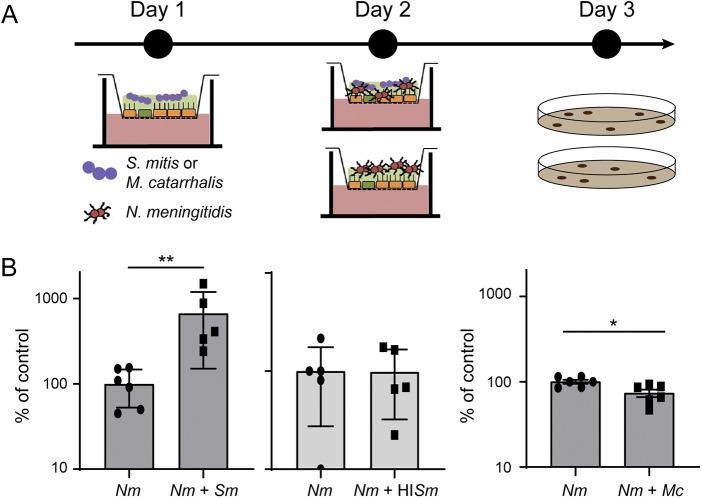
Coinfection with S. mitis and M. catarrhalis. (A) Schematic representation of the protocol followed for coinfection. (B) On day 1, cells were infected with 10^5^ S. mitis (*Sm*), heat-inactivated S. mitis (HI*Sm*), or M. catarrhalis (*Mc*). On day 2, cells were then infected with 10^6^ meningococci (N. meningitidis [*Nm*]). On day 3, bacteria were collected, and the number of CFU were determined. CFU of meningococci after 24 h of coculture were expressed as mean percentage of the control experiment ± SEM (CFU of meningococci in monoculture). Statistical analysis was performed by Student’s *t* test on at least five filters from three independent experiments. Values that are statistically significantly different are indicated by asterisks as follows: **, *P* < 0.01; * *P* < 0.05.

We next aimed at studying the interplay between N. meningitidis and other bacterial species in this model. Among species known to colonize the human nasopharynx, we selected Streptococcus mitis and Moraxella catarrhalis that are classically recovered from normal nasopharyngeal mucosa in adults and children, respectively (39–41). We first infected Calu-3 cells, cultured in AIC on a 0.4-μm-pore membrane, with 1 × 10^5^ S. mitis, or heat-inactivated S. mitis or M. catarrhalis. Both living bacteria were able to survive in the mucus of Calu-3 cells, but we did not detect proliferation 48 h after infection (S. mitis, inoculum, 3.08 × 10^5^ ± 0.8× 10^5^, 48 h, 2.7 × 10^5^ ± 1.01 × 10^5^; M. catarrhalis, inoculum, 4.12 × 10^5^ ± 1.9 × 10^5^, 48 h, 6.07 × 10^5^ ± 2.38 × 10^5^). We then infected Calu-3 cells colonized by heat-inactivated S. mitis or living S. mitis and M. catarrhalis with 1 × 10^6^ meningococci (wild-type 2C4.3 strain) for 24 h (Fig. 6). For a control, naive uninfected Calu-3 cells were infected by N. meningitidis. Our results showed that the coinfection with S. mitis significantly improved meningococcal colonization by 6.7-fold, while heat-inactivated S. mitis or living M. catarrhalis had no effect (Fig. 6). Interestingly, the positive effect of the S. mitis-N. meningitidis coinfection on the growth of meningococci appeared to be specific to the AIC model. A 24-h broth coculture of 1 × 10^5^ S. mitis and 1 × 10^6^ N. meningitidis revealed a slight decrease in meningococcal growth (Fig. S4A). Overall, these results support the hypothesis that N. meningitidis growth in AIC may be facilitated by other bacteria of the nasopharyngeal niche.

10.1128/mSphere.00494-19.4 FIG S4 Coculture of N. meningitidis with S. mitis in broth. BHI broth cultures with N. meningitidis (*Nm*) and S. mitis (*Sm*) or N. meningitidis alone were grown for 24 h, and N. meningitidis CFUs were determined. The number of meningococci after 24 h of growth was expressed as mean percentage of the value for the control experiment ± SEM. The control was meningococci grown in monoculture. Statistical analysis was performed by Student’s *t* test on four wells from two independent experiments. Values that are statistically significantly different are indicated by asterisks as follows: ****, *P* < 0.0001; **, *P* < 0.01. Download FIG S4, TIF file, 0.2 MB. Copyright © 2019 Audry et al. 2019 Audry et al. This content is distributed under the terms of the Creative Commons Attribution 4.0 International license.

Unlike M. catarrhalis, S. mitis is able to modify the mucus, for instance by hydrolysis of glycans, which are very abundant on mucin proteins (45). Hydrolysis of the carbohydrates of mucins might thus provide an additional source of carbon and nutrient for meningococci. For a marker of mucus modification by living S. mitis, we investigated the glycosylation profiles of mucins by mass spectrometry after infection or coinfection of Calu-3 cells with S. mitis, N. meningitidis, or M. catarrhalis (Table 1; see also Table S1 in the supplemental material). We observed a moderate oversialylation of mucins after colonization with N. meningitidis or M. catarrhalis, since 59% or 62.5% of the oligosaccharides detected, respectively, were sialylated compared to 36.2% in noninfected cells. The glycosylation profile of mucins has changed dramatically after the addition of S. mitis (Table 1 and Table S1). We noticed a global simplification of the O-glycans, due to dramatic desialylation of mucins probably leading to the release of sialic acid. Infection with heat-inactivated streptococci did not alter the glycosylation profile of mucins (Table S1). This confirms that S. mitis is capable of modifying the Calu-3 mucus and, at least, hydrolyzing mucins’ O-glycans, a process that correlates with increased N. meningitidis growth.

**TABLE 1:**
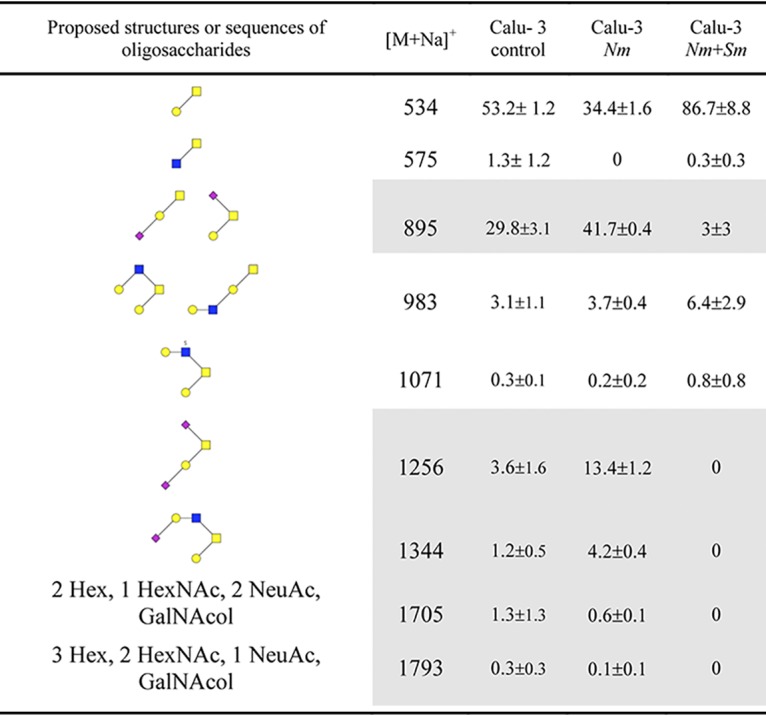
Proposed structures or sequences of oligosaccharides and glycosylation profiles of mucins on sialylated oligosaccharides and their nonsialylated forms identified on Calu-3 mucins^*a*^

a Proposed structures or sequences of oligosaccharides and glycosylation profiles of mucins on sialylated oligosaccharides and their nonsialylated forms identified on Calu-3 mucins before (control) and after infection with N. meningitidis (*Nm*) or coinfection with N. meningitidis and S. mitis (*Nm*+*Sm*). For the proposed structures, sugar types are represented by symbols as follows: blue squares, GlcNAc; yellow squares, GalNac; yellow circles, Gal; small red diamonds, Neu5Ac. The relative percentage of each oligosaccharide was calculated based on the integration of peaks on MS spectra. Two independent experiments of five different filters were studied in bulk. Results are presented as the mean relative percentage of each oligosaccharide ± SEM. A complete list of oligosaccharides is presented in Table S1 in the supplemental material. Sialylated glycans are indicated by gray shading.

10.1128/mSphere.00494-19.5 TABLE S1 Oligosaccharides identified on Calu-3 mucins. Download Table S1, DOCX file, 0.1 MB. Copyright © 2019 Audry et al. 2019 Audry et al. This content is distributed under the terms of the Creative Commons Attribution 4.0 International license.

## DISCUSSION

In this work, we adapted an experimental model, based on Calu-3 cells cultured in AIC, to study the behavior of N. meningitidis in the mucus of the respiratory tract. We have shown that meningococci are trapped in the mucus layer where bacteria were likely to be protected from desiccation and certainly gained access to nutrients. We found no evidence of active passage of N. meningitidis through the epithelial layer, and we observed that type IV pili were not important for growth or motility/mobility in this model. Similarly, we showed that virulence factors were poorly expressed in this model compared to culture in broth. Strikingly, this suggests commensal-like behavior of N. meningitidis, meaning that bacteria benefits from the cell layer/mucus layer without damaging it. This hypothesis is supported by the poor cytokine response observed 24 h after infection. We took advantage of this model to investigate the effect of other bacteria on the growth of N. meningitidis. We have shown that S. mitis, which is able to hydrolyze glycans, facilitates the growth of meningococci in a coinfection protocol.

Most studies aimed at determining the behavior of meningococci on epithelial cells has been done with cells that have been cultured and infected in LCC. Although these investigations provided a comprehensive description of the interaction between N. meningitidis and human epithelial cells, the scientific community has not been able to agree on the question of how and when meningococci cross the nasopharyngeal epithelium. During LCC infections, bacteria easily proliferate in the cell culture medium that contains amino acids, carbon source, and protein extracts. This allows N. meningitidis to grow and eventually cover the Calu-3 cell layer almost completely. When we infected Calu-3 cells cultured in AIC for 2 weeks, we observed a sixfold decrease in the total number of CFU per filter 24 h after infection. The bacteria were mainly found in the mucus where they organized into small groups of living and dying meningococci. As a consequence, bacteria rarely interact with human cells, and we have barely found N. meningitidis in direct contact with the plasma membrane of these cells. In view of this result, we asked whether N. meningitidis can cross the epithelial layer grown in AIC. After infection of cells cultured on 3-μm-pore membranes, we detected bacteria in the basal chamber of only two out of eight wells for the highest inoculum (10^4^ CFU) and no contaminated chambers for the lowest (10^2^ CFU). We then treated Calu-3 cells with IL-4 or IL-13, two cytokines that induce TEER decrease and induce mucus production, for 24 h (44). As expected, these two cytokines led to a reduction in TEER. However, this was not followed by an increase in the translocation of bacteria through the epithelial layer. All these results suggested that the traversal observed in this experiment was only stochastic and probably due to the heterogeneity of the mucus layer on the surfaces of the wells. To support this hypothesis, we have never observed meningococci of strain 2C4.3, inside or outside the cells, and in the vicinity of the porous membrane. Nevertheless, it will be interesting to evaluate the behavior of other isolates of N. meningitidis, especially strains of other serotypes such as MC58 or Z5463.

These results suggest that N. meningitidis growing in the mucus of epithelial cells did not alter the epithelial layer during the course of the experiment. We therefore studied the production of cytokines by epithelial cells grown in AIC or in LLC. Strikingly, we observed that three major inflammatory cytokines (IL-6, TNF-α, and IL-1β) were produced less during infection in AIC than in LCC. The two anti-inflammatory cytokine IL-10 and IL-4 were also produced less after infection, suggesting an overall reduction in the cytokine response during infection in AIC. However, after infection, Calu-3 cells secreted IL-12 and IFN-γ to the same extent whether they were infected in AIC or LCC. IFN-γ and IL-12 are known to be associated with macrophage and dendritic cell responses, although there is evidence that epithelial cells produce these cytokines after infection with microbes (46, 47). IFN-γ has pleiotropic effects on the epithelial cells of the respiratory tract. This cytokine has been shown to reduce MUC5AC expression, which may lead to a decrease in the barrier property of the respiratory mucus (48). In the meantime, IFN-γ induces the expression of CEACAM receptors (49) that are the receptors for the meningococcal adhesin Opa, known to be involved in the internalization of bacteria. Conversely, IFN-γ may promote the barrier function of lung epithelial cells (44). Finally, we did not detect IL-8 secretion after infection of Calu-3 cells. In our cytokine assay, AIC cultured cells were generally less reactive than cells that have been cultured in LCC, indicating that the mucus layer is likely to protect cells and retain pathogen-associated molecular patterns, resulting in a reduction in the innate immune response. However, our model, which did not include immune cells, gives only a partial overview of the innate immune response in the nasopharynx.

We observed that the expression of virulence factors by N. meningitidis varied according to growth status and that three virulence genes *pilE*, *mtrC*, and *fhbp* were significantly silenced during infection in AIC. The expression of *pilE* during infection in the AIC model appeared to be similar to that observed during the stationary phase of growth in liquid culture. This was correlated with the absence of a role for type IV pili during colonization of Calu-3 cells. Although most of the meningococcal strains found *in vivo* were piliated, the role of type IV pili during growth in the mucus was probably not related to motility and/or interaction with epithelial cells. However, we could not exclude an interaction between type IV pili and mucins. Conversely, the expression of *fhbp* during infection in AIC was dramatically decreased compared to the exponential and stationary phase of growth in broth. Interestingly, fHBP (factor H binding protein) is a key virulence factor of N. meningitidis (50) necessary for binding to human factor H and that inhibits the host alternative complement pathway. The role of fHBP in the respiratory tracts is not clear. While the respiratory mucus contains complement components (51, 52), the bactericidal activity of the complement is not clearly defined against N. meningitidis. For instance, acapsulated strains are regularly recovered by swabbing, whereas an active complement system should have eliminated these strains. It was therefore not surprising to observe the lack of regulation of *ctrA* gene expression between the different conditions tested. Based on our results, and in the context of the MenB vaccine, it may be important to further investigate the expression of *fhbp* in the context of respiratory mucus. Finally, we showed that *lbpA*, *tbpA*, and especially *fetA* were highly expressed in AIC, confirming the low concentration of free iron in this model.

The nasopharynx is colonized by six main genera: *Haemophilus*, *Streptococcus*, *Moraxella*, *Staphylococcus*, *Alloiococcus*, and *Corynebacterium* (40, 41). The impact of the microbiota on the growth, survival, and expression of N. meningitidis virulence factor is not yet known. Here, we have used the AIC model to address the impact on N. meningitidis growth of the colonization by two of these bacteria, Streptococcus mitis or Moraxella catarrhalis, that are typical colonizers of the human nasopharynx (39–41). We coinfected Calu-3 cells with S. mitis or M. catarrhalis and meningococci. Interestingly, we observed that S. mitis promoted meningococcal growth 24 h after infection. This result was not expected, since it is known that the pyruvate oxidase (SpxB) of *Streptococcaceae* produces a large amount of hydrogen peroxide and inhibits the growth of N. meningitidis in broth (53). Okahashi et al. have shown that S. mitis also expresses SpxB, which may be deleterious for Calu-3 cells (54). We therefore studied the growth of cocultured meningococci with S. mitis in broth (see Fig. S4 in the supplemental material). As described, a high ratio of S. mitis killed meningococci, while a ratio of 1 S. mitis per 10 N. meningitidis is sufficient to halve the total number of meningococci after 1 day of coculture. Conversely, in AIC, S. mitis promotes the growth of N. meningitidis, suggesting that S. mitis was less active against meningococci in AIC conditions. In addition, our glycomic analysis indicated that S. mitis is capable of hydrolyzing mucin O-glycans, while N. meningitidis is not. This was expected, since S. mitis is known to express many glycosyl hydrolases (45). Since sialic acids were released from the O-glycans, we assessed whether this could provide a growth advantage for N. meningitidis. As anticipated, meningococci were unable to grow in the presence of sialic acid as the sole carbon source in broth, and the addition of sialic acid in the mucus of Calu-3 cells was not sufficient to enhance the growth of meningococci (data not shown). However, it can be hypothesized that S. mitis might increase the concentration of other nutrients that may be metabolized by N. meningitidis, inhibit an antimicrobial peptide, or scavenge nutrients that N. meningitidis can then metabolize.

Altogether, our results have shown that infection of mucus-producing cells in AIC is different from that of conventional experiments performed in LCC. While the latter experiments have investigated the interaction of N. meningitidis with epithelial cells, which is likely to occur after substantial inflammation or mechanical breach in the mucus layer, our present study emphasizes that N. meningitidis is certainly trapped in the mucus layer and rarely interacts with human cells while the host response is less pronounced. Further work will be needed to better understand how N. meningitidis regulates its virulence factors and cohabits with other bacterial species in the mucus.

## MATERIALS AND METHODS

### Bacterial strains and growth conditions.

Neisseria meningitidis NEM 8013 (2C4.3), a piliated capsulated serogroup C strain, and its isogenic nonadhesive PilE defective mutant (Δ*pilE*) were used in this study (27).

Streptococcus mitis strain B26E10 (referenced as 0902 230473 in the Necker Hospital collection) and Moraxella catarrhalis strain B18F4 (referenced as B18F4 in Necker Hospital collection) were isolated from a patient in the Necker hospital (Paris). Except for the S. mitis strain grown on chocolate agar polyvitex plates, all strains were grown on brain hear infusion (BHI) agar plates supplemented with 5% horse serum at 37°C in a 5% CO_2_ incubator. The following antibiotics and concentrations were used: kanamycin at 100 μg/ml, vancomycin at 20 μg/ml, and polymyxin at 15 μg/ml.

### Cell culture.

Calu-3 epithelial cells (ATCC HTB-55) were maintained in optiMEM medium (Life Technologies) supplemented with 5% fetal bovine serum, HEPES, minimum amino acid solution, and penicillin-streptomycin antibiotics. Cells were grown in a 5% CO_2_ incubator at 37°C. Cells were grown on polyester 0.4-μm-pore membrane cell culture filter (Transwell; Corning). For traversal assays, 3-μm-pore membranes were used. Prior to cell seeding, the filter’s membranes were coated with type IV human placenta collagen (Sigma) for 24 h. Cells (3 × 10^5^) were seeded onto the apical side of membranes and were maintained in 200 μl of culture medium in the apical chamber and 1.2 ml in the basal chamber. In air interface culture (AIC) conditions, the apical culture medium was removed after 5 days, and cells were allowed to grow at the air interface for 3 to 6 days (week 0) or for 14 to 17 days (week 2) when mucus was evenly distributed at the surfaces of cell layers. The thickness of the mucus was estimated on representative confocal images and analyzed using ImageJ. Liquid-covered layers were seeded and cultured 14 to 17 days as described above, except that the apical media were maintained all along. The transepithelial electrical resistance (TEER) across air interface culture was measured with a Millicell Voltohmmeter (Millipore). Notably, the barrier function of the Calu-3 cell layer that had been grown on a 3-μm-pore membrane was decreased, as indicated by measurement of the transepithelial electrical resistance (TEER of 357 ± 19.83 Ω/cm^2^ using 0.4-μm-pore membrane; 258 ± 14.63 Ω/cm^2^ using 3-μm-pore membrane) (see Fig. S3C in the supplemental material). We estimated that Calu-3 cells accurately represent nasopharyngeal cells by comparing the glycosylation profiles of Calu-3 cell mucus and nasal mucus from the human donor. We have compared the repertoire of glycosylation of mucins secreted either by Calu-3 cells or human nasal mucosa (*n* = 5 donors) to demonstrate that major O-glycans carried by both set of mucins are similar (see Table S2 in the supplemental material).

10.1128/mSphere.00494-19.6 TABLE S2 Sialylated oligosaccharides and their nonsialylated forms identified on Calu-3 mucins or human nasal mucins. Download Table S2, DOCX file, 0.04 MB. Copyright © 2019 Audry et al. 2019 Audry et al. This content is distributed under the terms of the Creative Commons Attribution 4.0 International license.

### Infection.

**(i) Infection with N. meningitidis.** Two days before infection, antibiotics were removed from the culture media. On the day of infection, a suspension of bacteria from an overnight culture on an agar plate was diluted to a bacterial concentration of 5 × 10^7^ CFU/ml and cultured for 2 h at 37°C in optiMEM medium. The air interface culture cells were infected on the apical side with 10 μl of a bacterial suspension containing 10^6^ CFU per 10 μl unless specified otherwise. The next day, cells were collected by scraping and thoroughly vortexed, and then CFU were counted by plating serial dilutions onto agar plates. The same protocol was applied for liquid interface infections, except that a volume of 200 μl of bacterial suspension was used (10^6^ CFU per 200 μl). In order to separate bacteria present in the outer mucus fraction or in the cell-attached mucus fraction, an optiMEM−0.1% N-acetylcysteine solution was incubated for 15 min over the cells and harvested. This process was repeated three times, and CFU were counted in this outer mucus fraction by plating serial dilutions. Then for the cell-attached mucus fraction, the cells were scraped off, the collected cells were vortexed, and bacterial loads were assessed by plating CFU.

**(ii) Transmigration assay.** One day prior to infection with bacteria, human interleukin-4 (IL-4) and interleukin-13 (IL-13) were added in the basal chamber of Calu-3 cells cultured in AIC at 5 ng/ml each. Media were replaced immediately before infection, and IL-4 or IL-13 was added. At 4 and 24 h postinfection, media from the basal chambers containing untreated or treated cells were collected and centrifuged. Pellets were resuspended in 200 μl, and serial dilutions were cultured on agar plates.

**(iii) Coinfection and coculture.** At day 0, either S. mitis or M. catarrhalis strains grown on agar plates overnight were resuspended in optiMEM medium and cultured in optiMEM medium for 2 to 3 h at 37°C. After reaching the exponential growth phase, Calu-3 cells grown for 2 weeks in AIC were infected with 10 μl of bacterial suspension containing 1 × 10^5^ CFU. For the control filters, 10 μl of sterile medium was added over the cells. We also infected cells with heat-inactivated S. mitis boiled for 5 min at 95°C. At day 1, control and infected filters were infected with 1 × 10^6^ of N. meningitidis 2C4.3 strain as previously described. At day 2, bacteria were harvested by scraping and cultured on selective medium agar plates. The 2C4.3 strain was selected on vancomycin (20 μg/ml) when cocultured with S. mitis and on polymyxin (15 μg/ml) when cocultured with M. catarrhalis. During the assay, the cells were incubated at 37°C in a 5% CO_2_ incubator.

### Immunofluorescence assay.

**(i) Fixed cells.** For immunofluorescence assays, Calu-3 cells were grown in AIC and infected for 24 h. The filters were fixed with 4% paraformaldehyde for 1 h at room temperature, washed two times with phosphate-buffered saline (PBS) and permeabilized for 10 min with PBS−0.1% Triton X-100 and 10 min in PBS−0.1% bovine serum albumin (BSA)−0.1% X-100 Triton (staining buffer). The cells were then incubated with an anti-N. meningitidis strain 2C4.3 (anti-2C4.3) (and an anti-MUC5AC monoclonal antibody (clone 45M1; Life Technologies) in staining buffer for 2 h. After three washes in PBS, the filters were incubated with Alexa Fluor-conjugated secondary antibodies for 2 h. Nuclear DNA and actin were stained with 4′,6′-diamidino-2-phenylindole (DAPI) at 1 μg/ml and Alexa Fluor-conjugated phalloidin (Invitrogen), respectively. After several washes, the membranes were cut from the plastic support, and the coverslips were mounted in Mowiol for observation.

**(ii) Living cells.** Because the mucus could not be easily preserved through fixation, its production over time was monitored by imaging living cells labeled with an Alexa Fluor-conjugated dextran at 1 mg/ml (molecular weight [MW] of 10,000; Life Technologies) and Cell Trace Calcein Red Orange AM at 2.5 μM (Life Technologies) was used to stain the epithelium. The cell tracer was added in the basal chamber for 1 h, while the dextran solution was added on top of cells. Both solutions were removed and washed before confocal acquisition. During acquisition, the cells were maintained at 37°C under 5% CO_2_.

### Image analysis.

For three-dimensional (3D) reconstruction, image acquisition was performed on a laser-scanning confocal microscope (Leica TCS SP5). Fluorescence microscopy images were collected and processed using the Leica Application Suite AF Lite software. Each channel was adjusted for better visualization. 3D reconstruction, z-stack pictures, and posttreatment analyses were performed using Imaris software. Analysis of mucus thickness was performed using ImageJ.

### Electron microscopy.

**(i) Chemicals.** Crystalline osmium tetroxide (OsO_4_), sodium cacodylate, 25% glutaraldehyde, and Epon were from Euromedex (Souffelweyersheim, France). Hexamethyldisilazane (HMDS) was from Sigma-Aldrich (Lyon, France). Perfluoro compound FC-72 was from Fisher Scientific (Illkirch, France).

**(ii) Procedures.** All incubations were performed at room temperature. Whole inserts were fixed in 1% OsO_4_ diluted in FC-72 for 90 min, rinsed in FC-72 for 30 min, and fixed in 2.5% glutaraldehyde diluted in 0.1 M sodium cacodylate buffer (pH 7.4) for 90 min. The inserts were then rinsed in cacodylate buffer (twice for 30 min each time) and immersed in an ascending concentration of ethanol solutions (30%, 50%, 70%, 95%, 100%, 100%, and 100% for 10 min each time) for dehydration. For scanning electron microscopy (SEM), dehydration was completed by immersion in HMDS-ethanol (1/1, vol/vol) for 10 min and in HMDS for 10 min. After overnight air drying, each filter was removed from the insert using a small scalpel blade, placed on a piece of double-sided sticky tape on an aluminum stub, and sputter coated with Au/Pd. Images were acquired using a JEOL LV6510 (JEOL, Croissy-sur-Seine, France). For transmission electron microscopy (TEM), the inserts were immersed in an Epon-ethanol mixture of increasing Epon concentration (1/3 for 60 min, 1/1 for 60 min, 3/1 overnight) and finally in pure Epon (changed twice in 48 h). Each filter was removed from the insert and placed in an embedding capsule, with the cells facing down. After resin polymerization (2 h at 37°C and then 72 h at 60°C), the block was sectioned so as to produce sections of the cell layer. Ultrathin sections (80 nm) were stained in lead citrate and examined in a JEOL 100S (JEOL, Croissy-sur-Seine, France) at an accelerating voltage of 80 kV. Living bacteria were defined as circular and electron-dense cells.

### Desiccation assay.

The mucus was extracted from Calu-3 cells cultured in AIC for 2 weeks using 0.2% β-mercaptoethanol diluted in optiMEM (collection medium). Collection medium was added on top of the Calu-3 cells for 20 min and collected in a clean tube. This step was repeated three times. Mucus or fresh collection medium (as a control) was then applied to clean cell culture wells and dried overnight under a laminar flow hood. The next day, desiccated mucus or control medium were infected for 24 h with 5 × 10^6^ bacteria in 100 μl of cell culture medium. This allows for bacterial sedimentation and formation of a biofilm on the bottom of the well. The next day, culture supernatants were gently removed, and sedimented bacteria were exposed to desiccation for 0, 30, 60, and 120 min in a laminar flow hood. Bacteria were harvested and enumerated by quantitative culture on agar plates.

### Quantitative reverse transcription-PCR (RT-PCR).

**(i) RNA isolation.** Total RNA was isolated from N. meningitidis cultured at 37°C in optiMEM medium for 3 h (exponential growth phase) or overnight (stationary phase) or from infected cells (AIC or LCC [liquid-covered culture]) after 24 h. In those four conditions, bacteria were pelleted by centrifugation at maximum speed in a microcentrifuge for 2 min and quickly resuspended in cold TRIzol solution.

Samples were frozen and stored at –80°C. The samples were then treated with chloroform, and the aqueous phase was collected and used in the RNeasy clean-up protocol (Qiagen). RNA samples were incubated with turbo DNase (Invitrogen) for 1 h at 37°C before cleaning up on RNeasy minicolumn. Elution of RNA was done in nuclease-free water, and 1 μl of rRNasin (Promega) was added before storage.

**(ii) Retrotranscription.** cDNA synthesis reactions were carried out using the Lunascript RT Supermix kit (NEB), and 500 ng of RNA was used for each reaction.

**(iii) Quantitative RT-PCR method.** The 20-μl reaction mixture consisted of 10 μl of Luna Universal qPCR Master Mix, 0.5 μl of 10 μM of each primer, 1 μl of cDNA, and 8 μl of nuclease-free water. Pairs of primers were designed with Primer3Plus software. Gene expression levels were normalized by that of the housekeeping gene *pgm* (NMV_1606). Appropriate no-RT controls were conducted to ensure accuracy of the results.

### Cytokine quantification assay.

**(i) Sample preparations.** Calu-3 cells were grown on Transwells either in AIC or LCC for 2 weeks. For AIC, the cells were incubated at 37°C for 20 min with 100 μl of Ringer solution, and the apical supernatants were collected. This step was repeated once, and samples were kept on ice. For liquid-covered culture, 100 μl of medium in the apical chamber was collected, and the cells were washed with another volume of 100 μl of optiMEM medium. All samples were vortexed and centrifuged at 4°C for 5 min at maximum speed to eliminate bacteria and debris. Supernatants were harvested, snap-frozen in liquid nitrogen, and kept at –80°C before processing.

**(ii) Cytokine measurement.** Cytokines in cell supernatants were quantified by electrochemiluminescence multiplex assay kits from Meso Scale Discovery (Rockville, MD, USA). Briefly, 25 μl of supernatant was added to each well of 96-well multispot plates, and the assays were performed following the manufacturer’s instructions. Plates were read on the multiplexing imager Sector S600 (Meso Scale Discovery). All samples were measured in duplicate.

### Mucin glycosylation analysis.

**(i) Isolation and purification of mucins secreted by Calu-3 cells.** Cells were solubilized in 4 M guanidine chloride reduction buffer containing 10 mM dithiothreitol (DTT), 5 mM EDTA, 10 mM benzamidine, 5 mM *N*-ethylmaleimide, 0.1 mg/ml soy bean trypsin inhibitor, and 1 mM phenylmethanesulfonyl fluoride. Two milliliters of reduction buffer was added to each apical chamber and incubated overnight at room temperature. Cell suspensions were then gently agitated by pipetting, and each of the five filter suspensions per condition were pooled in a single aliquot. CsCl was added to an initial density of 1.4 g/ml, and mucins were purified by isopycnic density gradient centrifugation (Beckman Coulter LE80 K ultracentrifuge; 70.1 Ti rotor, 417,600 × *g* at 15°C for 72 h). Fractions of 1 ml were collected from the bottom of the tube and analyzed for periodic acid-Schiff (PAS) reactivity and density. The mucin-containing fractions were pooled, dialyzed against water, and lyophilized.

**(ii) Release of oligosaccharides from mucin by alkaline borohydride treatment.** Mucins were submitted to β-elimination under reductive conditions (0.1 M KOH and 1 M KBH_4_ for 24 h at 45°C), and the mixture of oligosaccharide alditols was dried on a rotavapor (Buchi) at 45°C. Borate salts were eliminated by several coevaporations with methanol before purification by cation exchange chromatography (Dowex 50 × 2, 200-400 mesh, H + form).

**(iii) Permethylation and mucin glycosylation analysis by MALDI-TOF MS.** Permethylation of the mixture of oligosaccharide alditols was conducted by the sodium hydroxide procedure of Ciucanu and Kerek (55). After derivatization, the reaction products were dissolved in 200 μl of methanol and further purified on a C_18_ Sep-Pak column (Waters, Milford, MA). Permethylated oligosaccharides were analyzed by matrix-assisted laser desorption ionization−time of flight mass spectrometry (MALDI-TOF MS) in a positive-ion reflective mode as [M+Na]^+^. Quantification through the relative percentage of each oligosaccharide was calculated based on the integration of peaks on MS spectra.

### Statistics.

Statistical analyses were performed using GraphPad Prism 8 software. One-way analysis of variance (ANOVA), Student’s *t* test, or Mann-Whitney U test was used in this study. In cases of unequal variance, a Welch ANOVA was applied instead of one-way ANOVA. *P* values of <0.05 were considered to indicate statistical significance. All images presented in this work were representative images.

10.1128/mSphere.00494-19.7 TABLE S3 Primers used in this study. Download Table S3, DOCX file, 0.02 MB. Copyright © 2019 Audry et al. 2019 Audry et al. This content is distributed under the terms of the Creative Commons Attribution 4.0 International license.

## References

[1] Join-Lambert O, Lecuyer H, Miller F, Lelievre L, Jamet A, Furio L, Schmitt A, Pelissier P, Fraitag S, Coureuil M, Nassif X 2013 Meningococcal interaction to microvasculature triggers the tissular lesions of purpura fulminans. J Infect Dis 208:1590–1597. doi:10.1093/infdis/jit301. 23840047

[2] Brandtzaeg P, van Deuren M 2012 Classification and pathogenesis of meningococcal infections. Methods Mol Biol 799:21–35. doi:10.1007/978-1-61779-346-2_2. 21993637

[3] Lecuyer H, Borgel D, Nassif X, Coureuil M 2017 Pathogenesis of meningococcal purpura fulminans. Pathog Dis 75:ftx027. doi:10.1093/femspd/ftx027. 28334263

[4] Capel E, Barnier JP, Zomer AL, Bole-Feysot C, Nussbaumer T, Jamet A, Lecuyer H, Euphrasie D, Virion Z, Frapy E, Pelissier P, Join-Lambert O, Rattei T, Bourdoulous S, Nassif X, Coureuil M 2017 Peripheral blood vessels are a niche for blood-borne meningococci. Virulence 8:1808–1819. doi:10.1080/21505594.2017.1391446. 29099305 PMC5810509

[5] Lecuyer H, Virion Z, Barnier JP, Matczak S, Bourdoulous S, Bianchini E, Saller F, Borgel D, Nassif X, Coureuil M 2018 An ADAM-10 dependent EPCR shedding links meningococcal interaction with endothelial cells to purpura fulminans. PLoS Pathog 14:e1006981. doi:10.1371/journal.ppat.1006981. 29630665 PMC5908201

[6] Bonazzi D, Lo Schiavo V, Machata S, Djafer-Cherif I, Nivoit P, Manriquez V, Tanimoto H, Husson J, Henry N, Chaté H, Voituriez R, Duménil G 2018 Intermittent pili-mediated forces fluidize Neisseria meningitidis aggregates promoting vascular colonization. Cell 174:143–155.e16. doi:10.1016/j.cell.2018.04.010. 29779947

[7] Simonis A, Schubert-Unkmeir A 2016 Interactions of meningococcal virulence factors with endothelial cells at the human blood-cerebrospinal fluid barrier and their role in pathogenicity. FEBS Lett 590:3854–3867. doi:10.1002/1873-3468.12344. 27498906

[8] Coureuil M, Lecuyer H, Bourdoulous S, Nassif X 2017 A journey into the brain: insight into how bacterial pathogens cross blood-brain barriers. Nat Rev Microbiol 15:149–159. doi:10.1038/nrmicro.2016.178. 28090076

[9] Cremers AJ, Zomer AL, Gritzfeld JF, Ferwerda G, van Hijum SA, Ferreira DM, Shak JR, Klugman KP, Boekhorst J, Timmerman HM, de Jonge MI, Gordon SB, Hermans PW 2014 The adult nasopharyngeal microbiome as a determinant of pneumococcal acquisition. Microbiome 2:44. doi:10.1186/2049-2618-2-44. 25671106 PMC4323220

[10] Wang H, Dai W, Feng X, Zhou Q, Wang H, Yang Y, Li S, Zheng Y 2018 Microbiota composition in upper respiratory tracts of healthy children in Shenzhen, China, differed with respiratory sites and ages. BioMed Res Int 2018:6515670. doi:10.1155/2018/6515670. 30013985 PMC6022278

[11] Esposito S, Principi N 2018 Impact of nasopharyngeal microbiota on the development of respiratory tract diseases. Eur J Clin Microbiol Infect Dis 37:1–7. doi:10.1007/s10096-017-3076-7. 28795339

[12] Biesbroek G, Tsivtsivadze E, Sanders EA, Montijn R, Veenhoven RH, Keijser BJ, Bogaert D 2014 Early respiratory microbiota composition determines bacterial succession patterns and respiratory health in children. Am J Respir Crit Care Med 190:1283–1292. doi:10.1164/rccm.201407-1240OC. 25329446

[13] Santee CA, Nagalingam NA, Faruqi AA, DeMuri GP, Gern JE, Wald ER, Lynch SV 2016 Nasopharyngeal microbiota composition of children is related to the frequency of upper respiratory infection and acute sinusitis. Microbiome 4:34. doi:10.1186/s40168-016-0179-9. 27364497 PMC4929776

[14] Ali MY 1965 Histology of the human nasopharyngeal mucosa. J Anat 99:657–672. 5857093 PMC1270703

[15] Freeman SC, Kahwaji CI 2018 Physiology, nasal. StatPearls Publishing, Treasure Island, FL. 30252342

[16] Fahy JV, Dickey BF 2010 Airway mucus function and dysfunction. N Engl J Med 363:2233–2247. doi:10.1056/NEJMra0910061. 21121836 PMC4048736

[17] Lillehoj EP, Kato K, Lu W, Kim KC 2013 Cellular and molecular biology of airway mucins. Int Rev Cell Mol Biol 303:139–202. doi:10.1016/B978-0-12-407697-6.00004-0. 23445810 PMC5593132

[18] Hoegger MJ, Awadalla M, Namati E, Itani OA, Fischer AJ, Tucker AJ, Adam RJ, McLennan G, Hoffman EA, Stoltz DA, Welsh MJ 2014 Assessing mucociliary transport of single particles in vivo shows variable speed and preference for the ventral trachea in newborn pigs. Proc Natl Acad Sci U S A 111:2355–2360. doi:10.1073/pnas.1323633111. 24474805 PMC3926068

[19] Ganz T 2002 Antimicrobial polypeptides in host defense of the respiratory tract. J Clin Invest 109:693–697. doi:10.1172/JCI15218. 11901174 PMC150915

[20] Cole AM, Dewan P, Ganz T 1999 Innate antimicrobial activity of nasal secretions. Infect Immun 67:3267–3275. 10377100 10.1128/iai.67.7.3267-3275.1999 PMC116505

[21] Brandtzaeg P 2009 Mucosal immunity: induction, dissemination, and effector functions. Scand J Immunol 70:505–515. doi:10.1111/j.1365-3083.2009.02319.x. 19906191

[22] Virji M, Alexandrescu C, Ferguson DJ, Saunders JR, Moxon ER 1992 Variations in the expression of pili: the effect on adherence of Neisseria meningitidis to human epithelial and endothelial cells. Mol Microbiol 6:1271–1279. doi:10.1111/j.1365-2958.1992.tb00848.x. 1353602

[23] Rudel T, van Putten JP, Gibbs CP, Haas R, Meyer TF 1992 Interaction of two variable proteins (PilE and PilC) required for pilus-mediated adherence of Neisseria gonorrhoeae to human epithelial cells. Mol Microbiol 6:3439–3450. doi:10.1111/j.1365-2958.1992.tb02211.x. 1362447

[24] Nassif X, Lowy J, Stenberg P, O’Gaora P, Ganji A, So M 1993 Antigenic variation of pilin regulates adhesion of Neisseria meningitidis to human epithelial cells. Mol Microbiol 8:719–725. doi:10.1111/j.1365-2958.1993.tb01615.x. 8332064

[25] Marceau M, Beretti JL, Nassif X 1995 High adhesiveness of encapsulated Neisseria meningitidis to epithelial cells is associated with the formation of bundles of pili. Mol Microbiol 17:855–863. doi:10.1111/j.1365-2958.1995.mmi_17050855.x. 8596435

[26] Merz AJ, Rifenbery DB, Arvidson CG, So M 1996 Traversal of a polarized epithelium by pathogenic Neisseriae: facilitation by type IV pili and maintenance of epithelial barrier function. Mol Med 2:745–754. doi:10.1007/BF03401658. 8972489 PMC2230138

[27] Pujol C, Eugene E, de Saint Martin L, Nassif X 1997 Interaction of Neisseria meningitidis with a polarized monolayer of epithelial cells. Infect Immun 65:4836–4842. 9353073 10.1128/iai.65.11.4836-4842.1997 PMC175694

[28] Edwards VL, Wang LC, Dawson V, Stein DC, Song W 2013 Neisseria gonorrhoeae breaches the apical junction of polarized epithelial cells for transmigration by activating EGFR. Cell Microbiol 15:1042–1057. doi:10.1111/cmi.12099. 23279089 PMC5584544

[29] Virji M, Makepeace K, Ferguson DJ, Achtman M, Moxon ER 1993 Meningococcal Opa and Opc proteins: their role in colonization and invasion of human epithelial and endothelial cells. Mol Microbiol 10:499–510. doi:10.1111/j.1365-2958.1993.tb00922.x. 7968528

[30] de Vries FP, van der Ende A, van Putten JP, Dankert J 1996 Invasion of primary nasopharyngeal epithelial cells by Neisseria meningitidis is controlled by phase variation of multiple surface antigens. Infect Immun 64:2998–3006. 8757826 10.1128/iai.64.8.2998-3006.1996 PMC174180

[31] Billker O, Popp A, Gray-Owen SD, Meyer TF 2000 The structural basis of CEACAM-receptor targeting by neisserial Opa proteins. Trends Microbiol 8:258–261. doi:10.1016/S0966-842X(00)01771-6. 10838580

[32] Sutherland TC, Quattroni P, Exley RM, Tang CM 2010 Transcellular passage of Neisseria meningitidis across a polarized respiratory epithelium. Infect Immun 78:3832–3847. doi:10.1128/IAI.01377-09. 20584970 PMC2937448

[33] Barrile R, Kasendra M, Paccani SR, Merola M, Pizza M, Baldari C, Soriani M, Arico B 2015 Neisseria meningitidis subverts the polarized organization and intracellular trafficking of host cells to cross the epithelial barrier. Cell Microbiol 17:1365. doi:10.1111/cmi.12439. 25801707

[34] Stephens DS, Hoffman LH, McGee ZA 1983 Interaction of Neisseria meningitidis with human nasopharyngeal mucosa: attachment and entry into columnar epithelial cells. J Infect Dis 148:369–376. doi:10.1093/infdis/148.3.369. 6413594

[35] Stephens DS, Whitney AM, Melly MA, Hoffman LH, Farley MM, Frasch CE 1986 Analysis of damage to human ciliated nasopharyngeal epithelium by Neisseria meningitidis. Infect Immun 51:579–585. 2867973 10.1128/iai.51.2.579-585.1986 PMC262381

[36] Stephens DS, Farley MM 1991 Pathogenic events during infection of the human nasopharynx with Neisseria meningitidis and Haemophilus influenzae. Rev Infect Dis 13:22–33. doi:10.1093/clinids/13.1.22. 1901998

[37] Read RC, Fox A, Miller K, Gray T, Jones N, Borrows R, Jones DM, Finch RG 1995 Experimental infection of human nasal mucosal explants with Neisseria meningitidis. J Med Microbiol 42:353–361. doi:10.1099/00222615-42-5-353. 7752215

[38] Kreft ME, Jerman UD, Lasic E, Hevir-Kene N, Rizner TL, Peternel L, Kristan K 2015 The characterization of the human cell line Calu-3 under different culture conditions and its use as an optimized in vitro model to investigate bronchial epithelial function. Eur J Pharm Sci 69:1–9. doi:10.1016/j.ejps.2014.12.017. 25555374

[39] Stearns JC, Davidson CJ, McKeon S, Whelan FJ, Fontes ME, Schryvers AB, Bowdish DM, Kellner JD, Surette MG 2015 Culture and molecular-based profiles show shifts in bacterial communities of the upper respiratory tract that occur with age. ISME J 9:1246–1259. doi:10.1038/ismej.2014.250. 25575312 PMC4409167

[40] Bogaert D, Keijser B, Huse S, Rossen J, Veenhoven R, van Gils E, Bruin J, Montijn R, Bonten M, Sanders E 2011 Variability and diversity of nasopharyngeal microbiota in children: a metagenomic analysis. PLoS One 6:e17035. doi:10.1371/journal.pone.0017035. 21386965 PMC3046172

[41] Teo SM, Mok D, Pham K, Kusel M, Serralha M, Troy N, Holt BJ, Hales BJ, Walker ML, Hollams E, Bochkov YA, Grindle K, Johnston SL, Gern JE, Sly PD, Holt PG, Holt KE, Inouye M 2015 The infant nasopharyngeal microbiome impacts severity of lower respiratory infection and risk of asthma development. Cell Host Microbe 17:704–715. doi:10.1016/j.chom.2015.03.008. 25865368 PMC4433433

[42] Etienne-Manneville S, Hall A 2001 Integrin-mediated activation of Cdc42 controls cell polarity in migrating astrocytes through PKCzeta. Cell 106:489–498. doi:10.1016/s0092-8674(01)00471-8. 11525734

[43] Merz AJ, Enns CA, So M 1999 Type IV pili of pathogenic Neisseriae elicit cortical plaque formation in epithelial cells. Mol Microbiol 32:1316–1332. doi:10.1046/j.1365-2958.1999.01459.x. 10383771

[44] Ahdieh M, Vandenbos T, Youakim A 2001 Lung epithelial barrier function and wound healing are decreased by IL-4 and IL-13 and enhanced by IFN-gamma. Am J Physiol Cell Physiol 281:C2029–C2038. doi:10.1152/ajpcell.2001.281.6.C2029. 11698262

[45] Derrien M, van Passel MW, van de Bovenkamp JH, Schipper RG, de Vos WM, Dekker J 2010 Mucin-bacterial interactions in the human oral cavity and digestive tract. Gut Microbes 1:254–268. doi:10.4161/gmic.1.4.12778. 21327032 PMC3023607

[46] Rouabhia M, Ross G, Page N, Chakir J 2002 Interleukin-18 and gamma interferon production by oral epithelial cells in response to exposure to Candida albicans or lipopolysaccharide stimulation. Infect Immun 70:7073–7080. doi:10.1128/iai.70.12.7073-7080.2002. 12438388 PMC133048

[47] Hadifar S, Behrouzi A, Fateh A, Khatami S, Rahimi Jamnani F, Siadat SD, Vaziri F 2019 Comparative study of interruption of signaling pathways in lung epithelial cell by two different Mycobacterium tuberculosis lineages. J Cell Physiol 234:4739–4753. doi:10.1002/jcp.27271. 30192006

[48] Oyanagi T, Takizawa T, Aizawa A, Solongo O, Yagi H, Nishida Y, Koyama H, Saitoh A, Arakawa H 2017 Suppression of MUC5AC expression in human bronchial epithelial cells by interferon-gamma. Allergol Int 66:75–82. doi:10.1016/j.alit.2016.05.005. 27324793

[49] Zhu Y, Song D, Song Y, Wang X 2019 Interferon gamma induces inflammatory responses through the interaction of CEACAM1 and PI3K in airway epithelial cells. J Transl Med 17:147. doi:10.1186/s12967-019-1894-3. 31072323 PMC6507156

[50] McNeil LK, Zagursky RJ, Lin SL, Murphy E, Zlotnick GW, Hoiseth SK, Jansen KU, Anderson AS 2013 Role of factor H binding protein in Neisseria meningitidis virulence and its potential as a vaccine candidate to broadly protect against meningococcal disease. Microbiol Mol Biol Rev 77:234–252. doi:10.1128/MMBR.00056-12. 23699256 PMC3668674

[51] Peters-Hall JR, Brown KJ, Pillai DK, Tomney A, Garvin LM, Wu X, Rose MC 2015 Quantitative proteomics reveals an altered cystic fibrosis in vitro bronchial epithelial secretome. Am J Respir Cell Mol Biol 53:22–32. doi:10.1165/rcmb.2014-0256RC. 25692303 PMC4566109

[52] Kulkarni HS, Liszewski MK, Brody SL, Atkinson JP 2018 The complement system in the airway epithelium: an overlooked host defense mechanism and therapeutic target? J Allergy Clin Immunol 141:1582–1586.e1. doi:10.1016/j.jaci.2017.11.046. 29339260 PMC5955701

[53] Pericone CD, Overweg K, Hermans PW, Weiser JN 2000 Inhibitory and bactericidal effects of hydrogen peroxide production by Streptococcus pneumoniae on other inhabitants of the upper respiratory tract. Infect Immun 68:3990–3997. doi:10.1128/iai.68.7.3990-3997.2000. 10858213 PMC101678

[54] Okahashi N, Sumitomo T, Nakata M, Sakurai A, Kuwata H, Kawabata S 2014 Hydrogen peroxide contributes to the epithelial cell death induced by the oral mitis group of streptococci. PLoS One 9:e88136. doi:10.1371/journal.pone.0088136. 24498253 PMC3909332

[55] Ciucanu I, Kerek F 1984 A simple and rapid method for the permethylation of carbohydrates. Carbohydr Res 131:209–217. doi:10.1016/0008-6215(84)85242-8.

