# Supplementary figures and images for: Airway Mucus Restricts Neisseria meningitidis Away from Nasopharyngeal Epithelial Cells and Protects the Mucosa from Inflammation
Source: mSphere. 2019 Dec 4;4(6):e00494-19. doi: 10.1128/mSphere.00494-19 (PMC6893211; doi:10.1128/mSphere.00494-19)

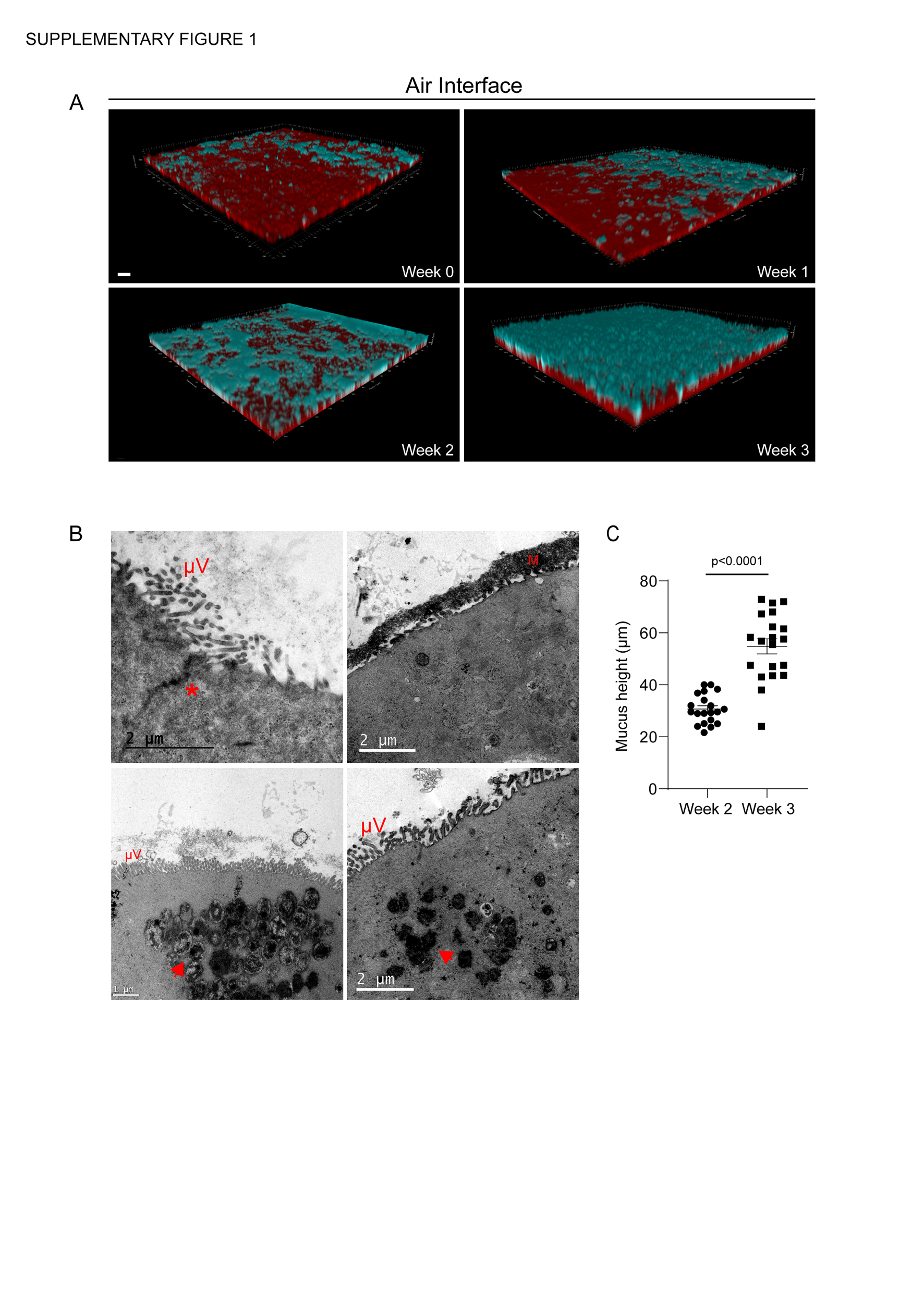

Supplement: FIG S1 [file mSphere.00494-19-sf001.tif]

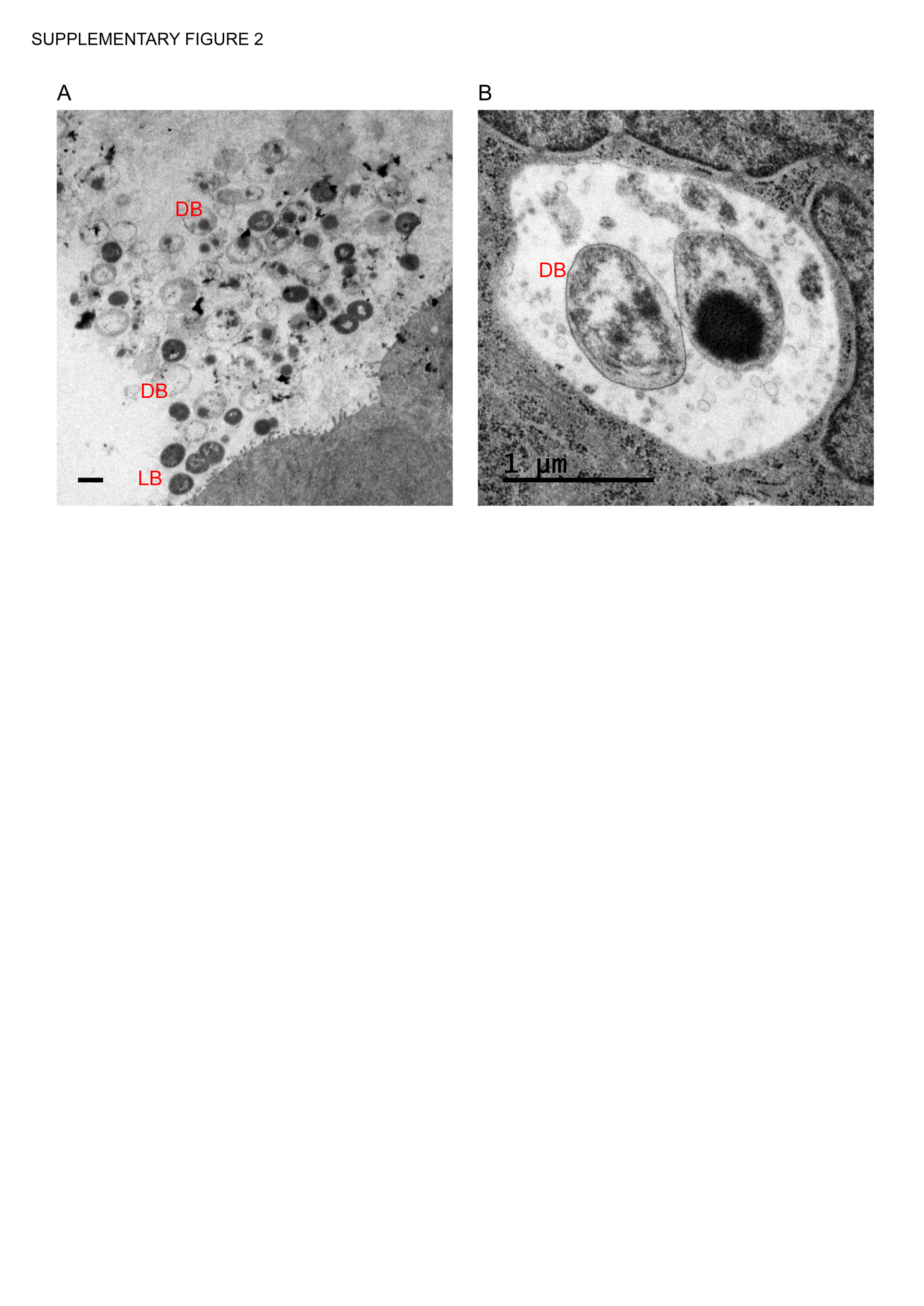

Supplement: FIG S2 [file mSphere.00494-19-sf002.tif]

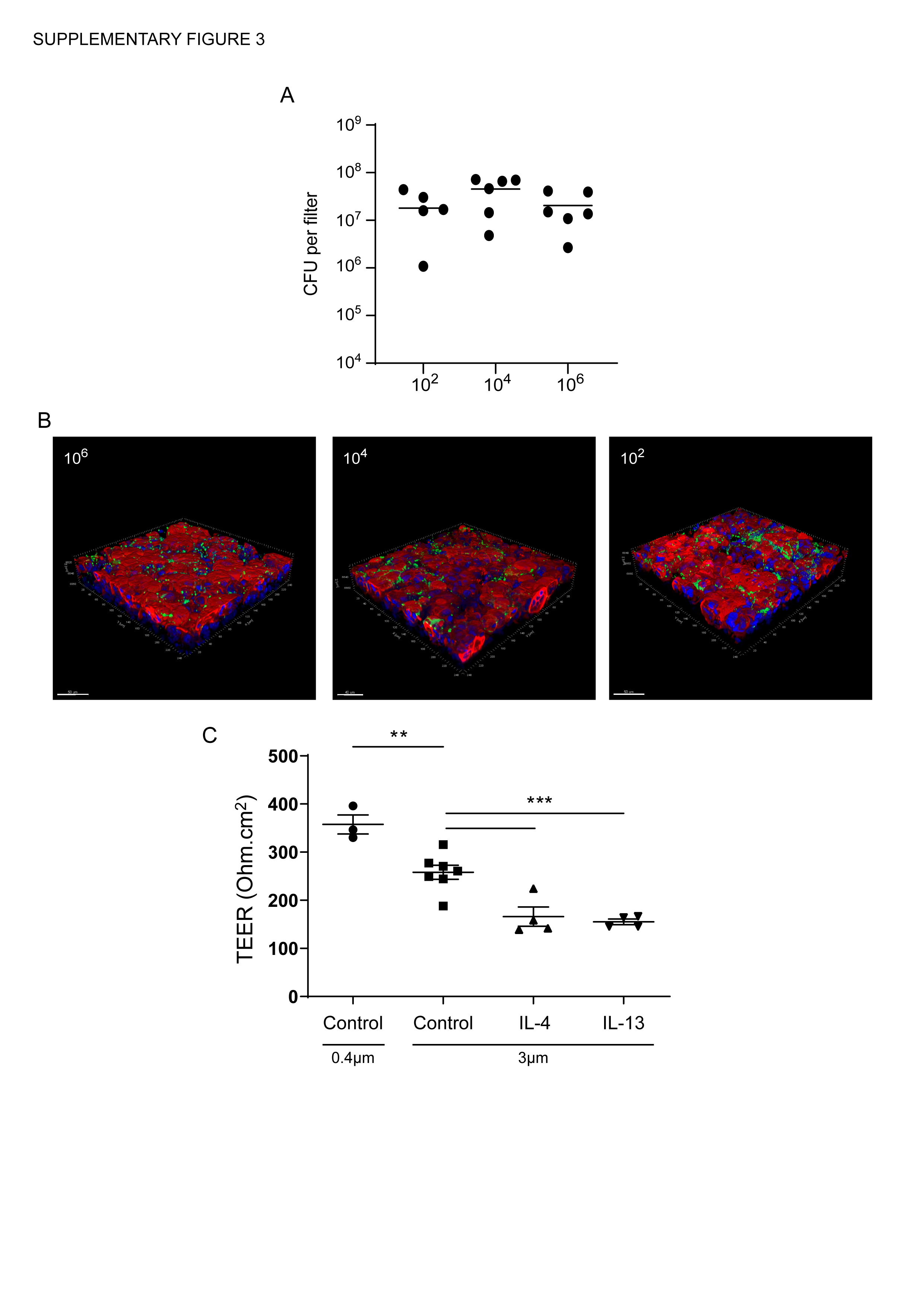

Supplement: FIG S3 [file mSphere.00494-19-sf003.tif]

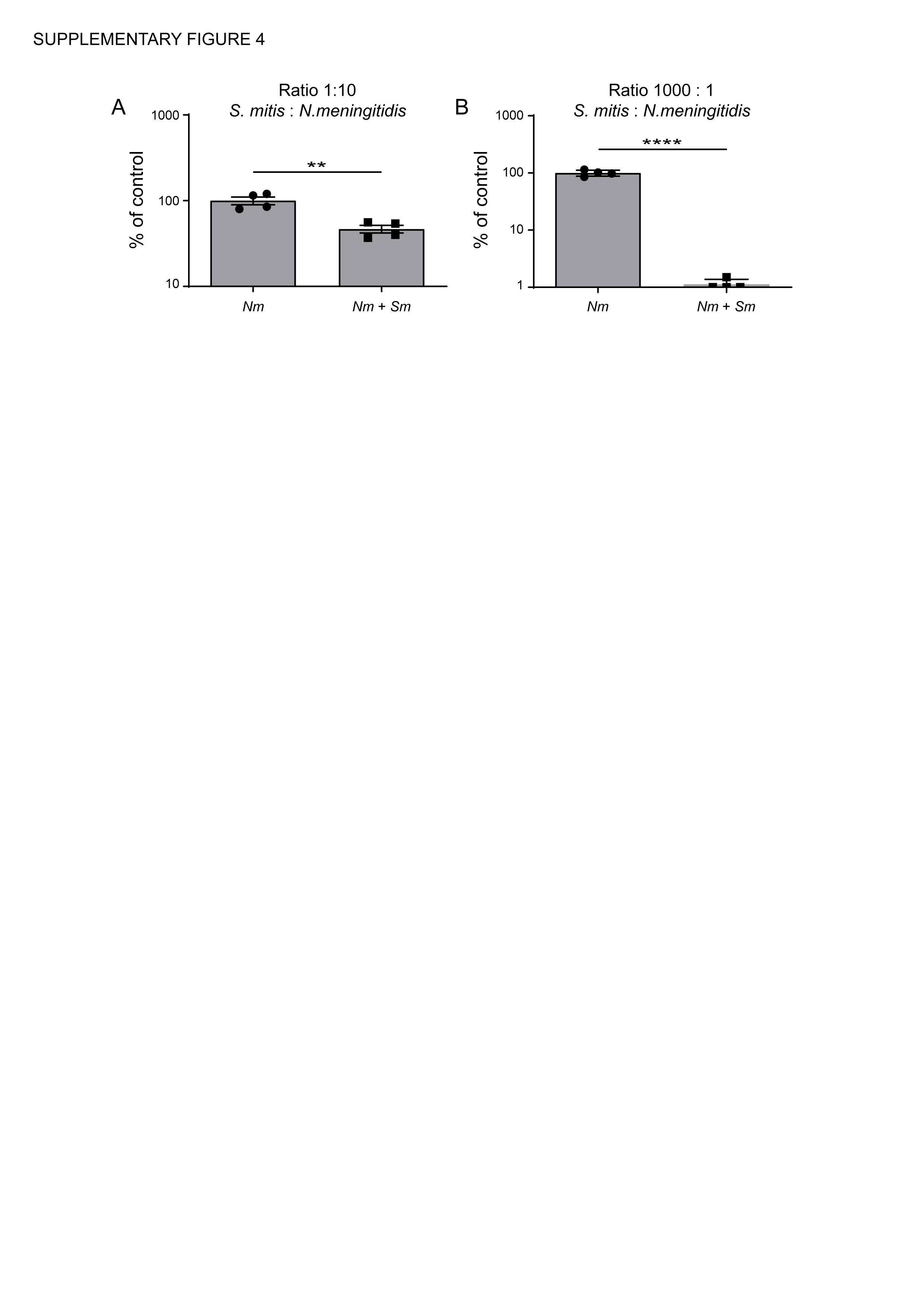

Supplement: FIG S4 [file mSphere.00494-19-sf004.tif]
