# Supplementary material for: Airway Mucus Restricts Neisseria meningitidis Away from Nasopharyngeal Epithelial Cells and Protects the Mucosa from Inflammation
Source: mSphere. 2019 Dec 4;4(6):e00494-19. doi: 10.1128/mSphere.00494-19 (PMC6893211; doi:10.1128/mSphere.00494-19)
Supplement: TABLE S2 [file mSphere.00494-19-st002.docx]

**Table S2.** Highlights on sialylated oligosaccharides, and their non sialylated form, identified on control Calu-3 mucins or human nasal mucins (n=5). The relative percentage of each oligosaccharide was calculated based on the integration of peaks on MS spectra. Two independent experiments of 5 different filters were studied in bulk. Results are presented as the mean relative percentage of each oligosaccharide ± SEM.

| Proposed structures or sequences of oligosaccharides | [M+Na]^+^ | Calu- 3 control | Nasal mucus |
| --- | --- | --- | --- |
| 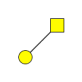 | 534 | 53,2± 1,2 | 24,9±3,2 |
| 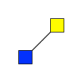 | 575 | 1,3± 1,2 | 2,2±1,5 |
| 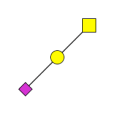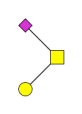 | 895 | 29,8±3,1 | 28±2,1 |
| 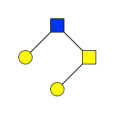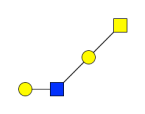 | 983 | 3,1±1,1 | 1,9±0,3 |
| 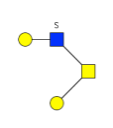 | 1071 | 0,3±0,1 | 0 |
| 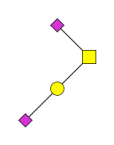 | 1256 | 3,6±1,6 | 5,4±1,7 |
| 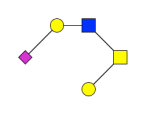 | 1344 | 1,2±0,5 | 2,7±0,9 |
| 2 Hex, 1 HexNAc, 2 NeuAc, GalNAcol | 1705 | 1,3±1,3 | 2,3±0,6 |
| 3 Hex, 2 HexNAc, 1 NeuAc, GalNAcol | 1793 | 0,3±0,3 | 0,1±0,1 |

Symbol for representation of sugar type ■ GlcNAc; GalNac; Gal; Fuc; ◆ Neu5Ac

Sialylated glycans are highlight in grey.
