# Supplementary material for: Airway Mucus Restricts Neisseria meningitidis Away from Nasopharyngeal Epithelial Cells and Protects the Mucosa from Inflammation
Source: mSphere. 2019 Dec 4;4(6):e00494-19. doi: 10.1128/mSphere.00494-19 (PMC6893211; doi:10.1128/mSphere.00494-19)
Supplement: TABLE S3 [file mSphere.00494-19-st003.docx]

**Table S3.** Primers used in this study.

*pilE* forward TTTGCGACTGTAACGCTTTG, reverse GCCATCCTTTTGGCTGAAGG

*porA* forward TCCCTTGAAAAACCATCAGG, reverse CAATTTCGGTCGTACTGTTT

*nadA* forward AAATTAGAAGCCGTGGCTGA, reverse TGCAGCGACAGCTTCGGCCT

*fhbp* forward CATACCGCCTTCAACCAACT, reverse GTTCGGCGGCGGCAAGCTCG

*nhbA* forward AAACGCCATTAGCCACATTC, reverse CCACGGCACCGAATATGCCA

*pgm* forward GCGAAGCCATAATGGAAAAA, reverse CTTTGCGGCAGGTTGTTTAA

*tonB* forward TCAGCAGCCTAAGGAAGAGC, reverse CTGCCTTCTCCGCGCCCCGT

*lbpA* forward GATAAGGCGGTGTTGTCGTT, reverse TGGAAGCATCGTAACCGAAG

*tbpA* forward GCAGTGGGGGATTCAGAGTA, reverse GGATGGGTATCCTCAACCGG

*fetA* forward CGGCAGAAAATAATGCCAAT, reverse GTGCCGTTGCCGCCGCCGAA

*ctrA* forward GTTTGGCGATGGTTATGCTT, reverse CGGCCTTTAATAATTTCCTG

*mtrC* forward CCGTACCGAACGATCAGAAT, reverse CTTCAGACCCGACGTAACAA

*opaB* forward CTTGTCCGCCATTTACGATT, reverse TGATACAAGCTTGCCTGGCT

*opaC* forward AACATCCGTACGCATTCCAT, reverse AAGCCGAGCGAGGAGACGGC
